# Nonflammable Lithium Metal Full Cells with Ultra-high Energy Density Based on Coordinated Carbonate Electrolytes

**DOI:** 10.1016/j.isci.2020.100844

**Published:** 2020-01-16

**Authors:** Sung-Ju Cho, Dae-Eun Yu, Travis P. Pollard, Hyunseok Moon, Minchul Jang, Oleg Borodin, Sang-Young Lee

**Affiliations:** 1 Department of Energy Engineering, School of Energy and Chemical Engineering, Ulsan National Institute of Science and Technology (UNIST), Ulsan 44919, Korea; 2 Battery Science Branch, Energy and Biomaterials Division, Sensor and Electron Devices Directorate, U.S. Army Research Laboratory, Adelphi, MD 20783, USA; 3 Battery R&D Center, LG Chem., Daejeon 34122, Korea

**Keywords:** Electrochemical Energy Storage, Electrochemical Materials Science, Electrochemical Energy Engineering

## Abstract

Coupling thin Li metal anodes with high-capacity/high-voltage cathodes such as LiNi_0.8_Co_0.1_Mn_0.1_O_2_ (NCM811) is a promising way to increase lithium battery energy density. Yet, the realization of high-performance full cells remains a formidable challenge. Here, we demonstrate a new class of highly coordinated, nonflammable carbonate electrolytes based on lithium bis(fluorosulfonyl)imide (LiFSI) in propylene carbonate/fluoroethylene carbonate mixtures. Utilizing an optimal salt concentration (4 M LiFSI) of the electrolyte results in a unique coordination structure of Li^+^-FSI^−^-solvent cluster, which is critical for enabling the formation of stable interfaces on both the thin Li metal anode and high-voltage NCM811 cathode. Under highly demanding cell configuration and operating conditions (Li metal anode = 35 μm, areal capacity/charge voltage of NCM811 cathode = 4.8 mAh cm^−2^/4.6 V, and anode excess capacity [relative to the cathode] = 0.83), the Li metal-based full cell provides exceptional electrochemical performance (energy densities = 679 Wh kg_cell_^−1^/1,024 Wh L_cell_^−1^) coupled with nonflammability.

## Highlights

• Nonflammable, ultra-high-energy-density Li metal full cells are demonstrated.
• Thin Li metal anodes are coupled with high-capacity/high-voltage NCM811 cathodes.
• 4 M LiFSI in PC/FEC shows unique coordination structure of Li^+^-FSI^-^-solvent cluster.
• The coordinated carbonate electrolyte forms both stable SEI and CEI layers.

## Introduction

Li metal batteries (LMBs) have garnered substantial attention as an appealing next-generation energy storage system (i.e., beyond Li-ion batteries [LIBs]) owing to the use of Li metal anodes possessing a low redox potential (−3.04 V versus standard hydrogen electrode), high specific capacity (3,860 mAh g_Li_^−1^), and low density (0.534 g cm^−3^) (Albertus et al., 2017). However, poor electrochemical reliability and safety concerns associated with the use of Li metal anodes, including low cycling Coulombic efficiency and nonuniform growth of Li dendrites, pose serious impediments to the realization of viable LMBs. Enormous efforts have been undertaken to overcome these problems, most of which have focused on the structuring/engineering of the Li metal anode and its interfacial stability with electrolytes (Lin et al., 2017, Cheng et al., 2016).

**Figure 1:**
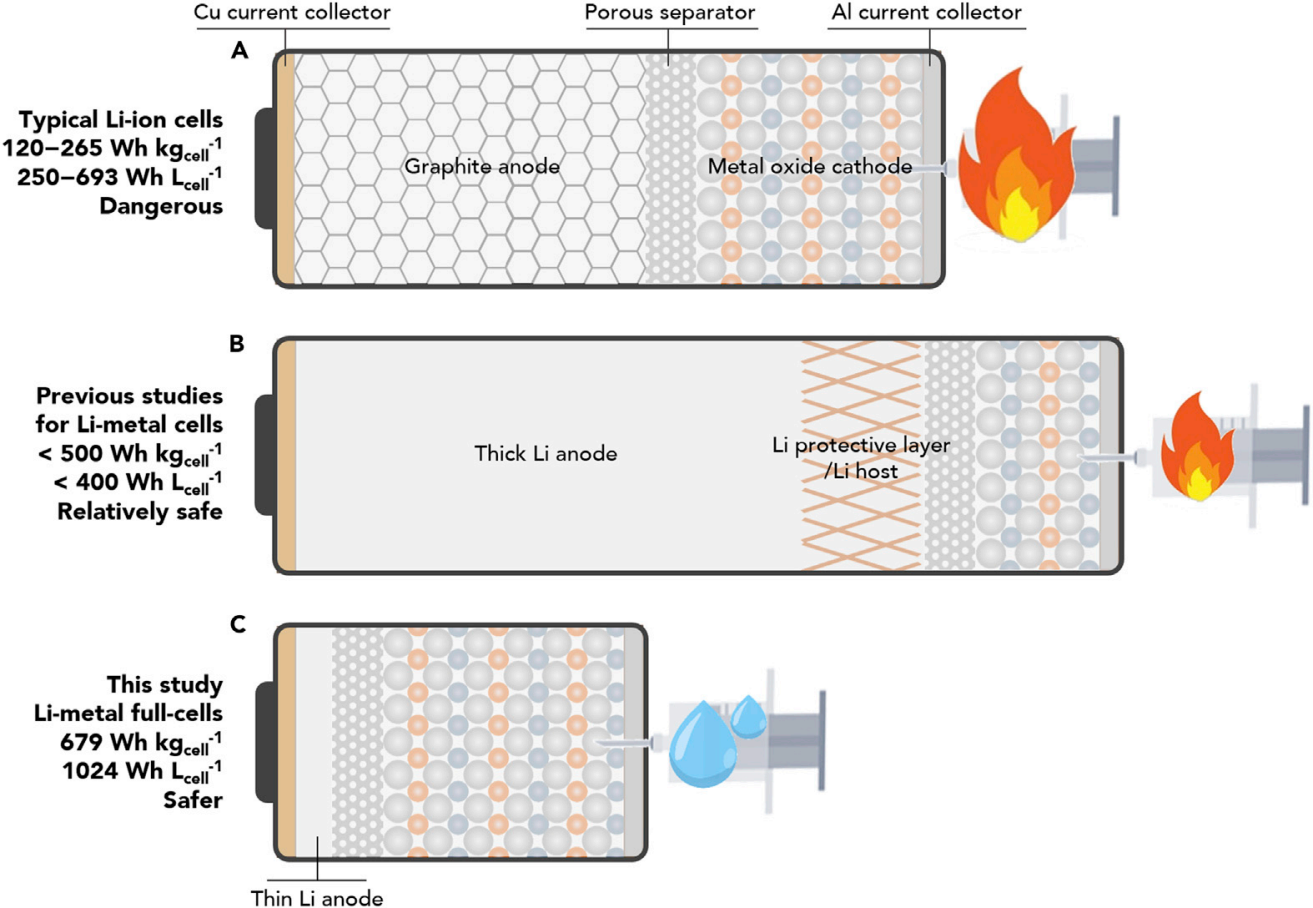
Conceptual Illustrations of Full Cell Structures and the Material Design of LIBs and LMBs (A) Conventional Li-ion full cell (Placke et al., 2017, Nishi, 2001, Broussely and Archdale, 2004). (B) Typical previously reported Li metal full cells (Jiao et al., 2018a, Jiao et al., 2018b, Zhao et al., 2018, Salitra et al., 2018, Gao et al., 2019). (C) Ultra-high-energy-density/nonflammable Li metal full cell reported here. The representative gravimetric/volumetric energy densities and safety behavior of the full cells are provided (the energy densities are estimated from the total weight and volume of the Li metal anode [excluding a Cu current collector], NCM811 cathode [excluding an Al current collector], and separator).

From a practical point of view, coupling thin Li metal anodes (Liu et al., 2019) with high-capacity/high-voltage cathodes (Jiao et al., 2018a, Jiao et al., 2018b) (ideally with nonflammable electrolytes) is essential to develop high-energy-density and safe LMBs. However, previous studies tended to employ thick Li metal anodes and low-areal-capacity cathodes, with little attention devoted to cell-based energy densities and safety issues (Zhao et al., 2018, Salitra et al., 2018). The importance of cell configuration and material design for practical Li metal full cells is conceptually illustrated, along with the comparative consideration of previous approaches (Placke et al., 2017, Nishi, 2001, Broussely and Archdale, 2004, Gao et al., 2019), in Figure 1. This demonstrates that electrolytes play a determinant role in both the electrochemical stability of the electrode-electrolyte interface and the safety of full cells.

The most widely investigated electrolytes for use with Li metal anodes are ether-based (e.g., 1,2-dimethoxyethane [DME], 1,3-dioxolane [DOL], etc.), but these have a low oxidation stability (thereby greatly restricting the cathode choice) and are typically highly flammable. In contrast, carbonate-based electrolytes (i.e., those commonly employed in commercial LIBs) have better stability with high-voltage cathodes, but suffer from poor electrochemical stability with Li metal anodes. Recently, some noteworthy electrolyte approaches based on the use of mixtures of (linear/cyclic) carbonate (Fan et al., 2018a), ether/carbonate (Fan et al., 2018b), and concentrated/multiple salts (Zheng et al., 2017, Ren et al., 2019) reportedly helped to resolve the electrolyte-electrode interfacial instabilities. However, the key requirements (specifically, Li metal anodes: ≤ 6 mAh cm^−2^ and ≤50 μm, cathodes: > 3 mAh cm^−2^ and >4.0 V, and safety) for practical Li metal-based full cells have not yet been met (Liu et al., 2018a, Liu et al., 2018b).

Here, we present highly coordinated nonflammable carbonate electrolytes based on lithium bis(fluorosulfonyl)imide (LiFSI) in propylene carbonate/fluoroethylene carbonate (PC/FEC = 93/7 v/v) mixtures as a new electrolyte strategy to enable ultra-high-energy-density and safer Li metal full cells. PC has been widely investigated as a thermally stable and even nonflammable organic solvent in LIB electrolytes, but it fails to form a stable solid electrolyte interface (SEI) layer on Li metal anodes (Takenaka et al., 2014). The present study, however, has identified an optimal salt concentration (4 M LiFSI), which exhibits a favorable coordination structure of Li^+^-FSI^−^-solvent clusters (e.g., (Li^+^)(FSI^−^)(PC)_1.6_(FEC)_0.18_), in which a small amount of FEC is added to further stabilize the electrode-electrolyte interface. In marked contrast to previous studies on PC-based electrolytes, this highly coordinated carbonate electrolyte forms stable interface layers on both thin Li metal anodes and LiNi_0.8_Co_0.1_Mn_0.1_O_2_ (NCM811) cathodes, as demonstrated here both experimentally and theoretically. The evaluated Li metal full cells (assembled with the low-capacity excess/thin Li metal anodes [4.0 mAh cm^−2^/35 μm], high-capacity/high-voltage NCM811 cathodes [4.8 mAh cm^−2^/4.6 V], and a highly coordinated nonflammable carbonate electrolyte [4 M LiFSI-PC/FEC]) have an exceptional electrochemical performance (in particular, energy densities = 679 Wh kg_cell_^−1^/1,024 Wh L_cell_^−1^ and 288 Wh kg_pouch_^−1^/437 Wh L_pouch_^−1^) coupled with high safety (nonflammability and normal cell operation even upon exposure to flame), traits that lie far beyond those reported for conventional battery technologies.

## Results and Discussion

### Interfacial Phenomena between the Coordinated Carbonate Electrolytes and Li Metal

**Figure 2:**
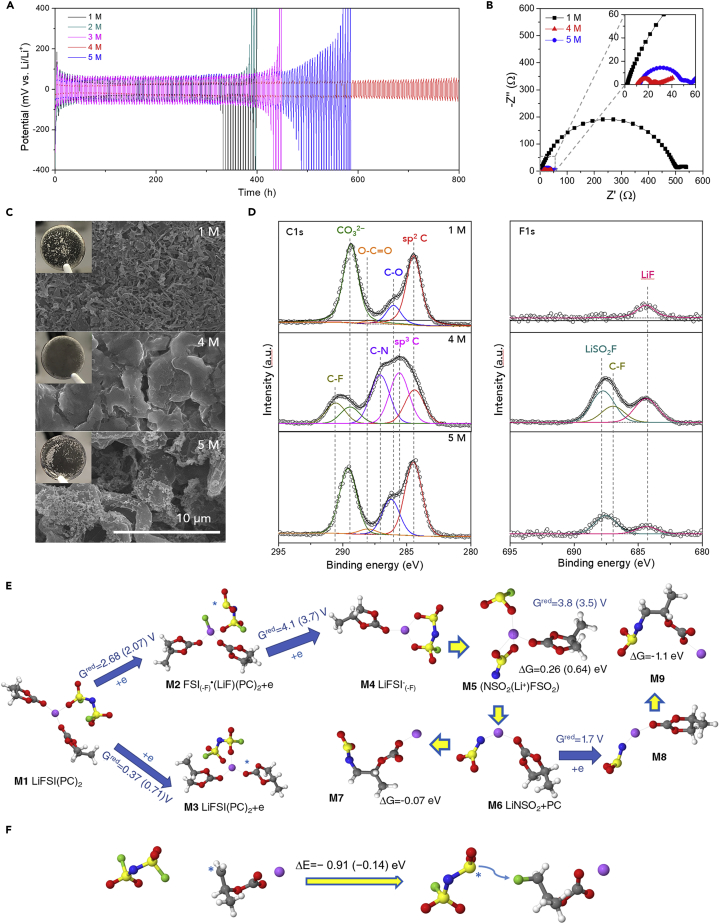
Interfacial Phenomena between Electrolytes and Li Metal (A) Li plating/stripping profiles of Li||Li symmetric cells with LiFSI-PC/FEC for different LiFSI concentrations at a current density of 0.2 mA cm^−2^. (B) EIS spectra of Li||Li symmetric cells with LiFSI-PC/FEC for different LiFSI concentrations. (C) Scanning electron microscopic images of Li metal surface after repeated Li plating/stripping test (20 cycles). (D) XPS C1s (left) and F1s (right) spectra of the SEI layers formed on the Li metal anodes. (E) Reduction and decomposition reactions for LiFSI(PC)_2_ complexes from B3LYP/6-31+G(d,p) and G4MP2 (in parentheses) QC calculations using PCM(acetone) implicit solvent models. (F) F-transfer reaction free energy from FSI^−^ to PC⋅ radical from B3LYP/6-31+G(d,p) and G4MP2 (in parentheses) calculations using PCM (acetone) implicit solvent models.

Li (20 μm)||Li (20 μm) symmetric cells containing LiFSI-PC/FEC electrolytes were examined by Li plating/stripping cycling tests at a current density of 0.2 mA cm^−2^ (Figure 2A). The cells with 1 and 2 M LiFSI-PC/FEC electrolytes had a large overpotential and eventually failed after 350 h. The cyclability of 3 M and 5 M LiFSI-PC/FEC electrolytes was improved, but they also had large overpotential after 420 and 500 h, respectively. This result reveals that the SEI layers formed by the 1, 2, 3, and 5 M LiFSI-PC/FEC electrolytes are not sufficiently stable and thus continuously consume electrolytes to form new SEIs, resulting in electrolyte depletion and accelerated growth of Li dendrites (Lu et al., 2015). In contrast, the cell with the 4 M LiFSI-PC/FEC electrolyte had a modest increase in overpotential and displayed reliable Li plating/stripping cyclability, without serious voltage fluctuation or internal short-circuit failure, over 800 h of cycling. Such a strong dependence on the salt concentration was further examined by electrochemical impedance spectroscopy (EIS) for 24 h under open-circuit voltage conditions, which provides information regarding the chemical stability of electrolytes with Li metal (Peled et al., 1997). The 4 M LiFSI-PC/FEC electrolyte has the lowest SEI resistance (R_SEI_ = 13.4 Ω) relative to the 1 and 5 M electrolytes (486.2 and 30.9 Ω, respectively) (Figure 2B), thus demonstrating that the 4 M LiFSI-PC/FEC electrolyte enables the formation of a stable SEI layer on Li metal, even though carbonate solvents are utilized.

The structural change on the Li metal surface after the repeated Li plating/stripping test (20 cycles) was investigated. A significant amount of needle-like Li dendrites, as well as dead (*i.e.*, electrically isolated) Li, was formed when using 1 M LiFSI-PC/FEC (Figure 2C). In addition, random dispersions of dark black Li deposits, often called *Elton's Gray Layer* (inset of Figure 2C) (Qian et al., 2015), were observed. The 4 M LiFSI-PC/FEC electrolyte, however, resulted instead in the formation of a nodular, dense/uniform surface morphology with a much lower surface area, thereby revealing the better interfacial compatibility with Li metal. In contrast, the result with the 5 M LiFSI-PC/FEC electrolyte consists of fractal Li dendrites on the underlying nodular morphology. Coulombic efficiency of Li plating/stripping was examined using a Li||Cu cell for the best performing 4 M LiFSI-PC/FEC electrolyte. Under a high Li utilization condition (areal capacity = 4 mAh cm^−2^ at a current density of 0.2 mA cm^−2^), a stable Coulombic efficiency (>99.5%) was maintained over 20 cycles (Figure S1).

To better elucidate this unusual interfacial behavior, the SEI layers on the Li metal were analyzed using X-ray photoelectron spectroscopy (XPS) (Figure 2D). The C1s spectra show that the SEI layer formed by 1 M LiFSI-PC/FEC has the expected typical carbonaceous species (sp^2^ C [284.5 eV], C−O [286.2 eV], O−C=O [287.8 eV], and CO_3_^2−^ [289.7 eV]). These organic species are known to originate from decomposition of carbonates (e.g., PC) (Li et al., 2015). In contrast and somewhat unexpectedly, 4 M LiFSI-PC/FEC exhibits two peculiar peaks tentatively assigned to C−N (287.0 eV) and C-F (290.8 eV) in the C1s spectra and also promotes the formation of inorganic species including LiSO_2_F (687.5 eV) and C−F (687.1 eV), as well as LiF (684.4 eV), in the F1s spectra. It is believed that LiSO_2_F and LiF arise from decomposition of LiFSI and FEC, in which LiSO_2_F is often produced in concentrated electrolytes (Gu et al., 2016). A comparison with the result using 5 M LiFSI-PC/FEC demonstrates that a salient feature of the SEI layer formed by 4 M LiFSI-PC/FEC is the presence of C−N and C−F, along with a larger proportion of inorganic species. Note that the C−N and C−F compounds have not been previously reported as SEI components of PC/FEC-based electrolytes, to the best of our knowledge. The origin of C−N and C−F compounds is theoretically identified in the following section. Based upon the results reported here, this unique SEI layer evidently enables the interfacial stabilization of Li metal, thereby suppressing the Li dendrite growth and the accompanied electrolyte consumption.

The coordination states of LiFSI-PC/FEC were qualitatively examined by Raman analysis (Figure S2). These support the formation of coordinated Li^+^-FSI^−^-solvent clusters (e.g., SSIP [solvent-separated ion pair], CIP [contact ion pair], and AGG (aggregate) distribution) (Seo et al., 2012) and the low proportion of free PC molecules at high LiFSI concentrations. Note that 4 M LiTFSI-PC/FEC corresponds to (Li^+^) (FSI^−^) (PC)_1.6_(FEC)_0.18_. Molecular dynamics (MD) simulations shown in Figures S3–S5 indicate a preference for a Li^+^ cation to be coordinated by a carbonyl oxygen of PC, followed by FEC carbonyl oxygens and FSI^−^ oxygens, resulting in a largely dissociated 1 M electrolyte, but electrolytes that are quite aggregated at the higher concentrations of 4 M and 5 M. For the 1 M concentration, a given Li^+^ cation is coordinated predominantly by 4 PCs; at 4 M it is equally coordinated by approximately two oxygens from PC and FSI^−^, whereas at 5 M the Li^+^ cation is coordinated mostly by 3 oxygens of FSI^−^ anions and 1 PC.

Based on this information regarding the Li^+^-FSI^−^-solvent clusters, electrolyte reduction and the initial stages of the SEI formation were examined using quantum chemistry (QC) calculations, as shown in Figures 2E and S6–S8. In the moderately concentrated regime, the (PC)_2_LiFSI CIP complex (M1 in Figure 2E) reduction leads to LiF formation at 2.68 V (versus Li/Li^+^) (M2 in Figure 2E), but requires a larger reorganization energy than for PC reduction, which occurs at a much lower potential of 0.71 V (versus Li/Li^+^) (M3 in Figure 2E) for CIP and at 0.5–0.6 V for SSIP Li^+^(PC) and larger aggregates (Figures S6 and S7A) (Von Cresce et al., 2012). Predicted reduction potentials agree well with linear sweep voltammetry (LSV) measurements (Figure S7E). Fast reduction, coupled with the F-transfer from FSI^−^ to the LiF surface covering the Li metal, was also observed in recent density functional theory (DFT) calculations of concentrated electrolytes (Alvarado et al., 2019). In some cases, F detachment is also observed during LiFSI reduction when no Li^+^ cation is present near this fluorine (Figures S7B and S8B). In a reducing environment, the detached F reduces to F^−^ (instead of participating in the C−F bond formation). The QC calculations, however, indicate that the C−F bond forms as a result of F transfer from FSI^−^ to Li(PC⋅) radical, as shown in Figure 2F in accord with the XPS results shown in Figure 2D. For a low 1 M LiFSI salt concentration, the Li(PC⋅) radical has a low probability of encountering an FSI^−^ anion and, thus, to form the C−F bond. At the highest LiFSI concentration of 5 M, most of the FSI^−^ anions have one or more Li^+^ cations near the FSI^−^ anions and there are fewer PC near Li^+^ cations (relative to the FSI^−^ anions) (Figure S8), thereby making the F-transfer as a result of Li_2_FSI reaction with the Li(PC⋅) radical less probable.

Next, we focused on understanding the mechanism of the C−N bond formation. The defluorined LiFSI radical (*i.e.*, LiFSI_(−F)⋅_) is expected to be readily reduced at the Li anode due to the high reduction potential of M2 to M4 reaction in Figure 2E. The reduced LiFSI_(-F)⋅_ decomposes by S−N bond breaking via the activated complex (NSO_2_^−^)Li^+^(SO_2_F^−^)PC (M5 in Figure 2E). This combination of reduction and S−N bond breaking is predicted to occur below 3.5 V (versus Li/Li^+^). Thus, this reaction is expected to occur when the SEI does not completely block electron tunneling from the Li metal. If the LiFSI_(−F)⋅_ radical reduction is assumed to occur before the S−N bond breaking, the later reaction is slightly endergonic (ΔG = 0.26 eV). Next, the LiNSO_2_ salt (complex M6) either reacts with PC to form complex M7 with a near-zero reaction energy or undergoes a second reduction at potentials below 1.7 V (versus Li/Li^+^) to form a more reactive radical M8 (LiNSO_2_⋅)^−^, which reacts with PC to form the N−C bond (M9 complex in Figure 2E). Alternatively, the C−N bond formation may occur as a result a PC⋅^−^ radical reaction with the (LiNSO_2_^−^)⋅ radical or reduction of the LiNSO_2_F(PC) complex, as shown in Figure S8. In contrast, when a low LiFSI salt concentration is used, the low number of CIP species and FSI^−^ anions near the anode instead results in Li^+^(PC) reduction being the dominant mechanism and no C−N bond formation is expected, in agreement with XPS results. Thus, MD simulations and QC calculations demonstrate that the 4 M LiFSI electrolyte concentration provides favorable conditions for the SEI containing both FSI and PC reduction product that are likely well mixed as indicated by formation of new compounds with C−N and C−F bonds that require contact of the reduced FSI and PC. Such homogeneous SEI is expected to be beneficial for stabilization of the Li metal anode.

### Effect of Coordinated Carbonate Electrolytes on Interfaces of NCM811 Cathodes

**Figure 3:**
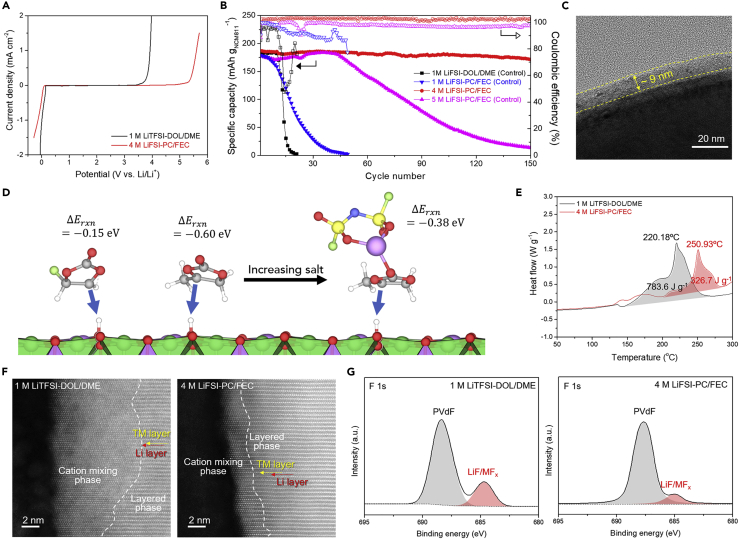
Effect of Coordinated Electrolytes on Interfaces of NCM811 Cathodes (A) Electrochemical stability window of 4 M LiFSI-PC/FEC and 1 M LiTFSI-DOL/DME (control) at a scan rate of 0.1 mV s^−1^. (B) Cycling performance of Li (4 mAh cm^−2^)||NCM811 (3.5 mAh cm^−2^) cells at a charge/discharge current density of 0.35 mA cm^−2^ (voltage range: 3.0–4.2 V). (C) High-resolution transmission electron microscopic image of NCM811 particles (after 150 cycles) in 4 M LiFSI-PC/FEC. (D) Reactivity of FEC, PC, and PC(LiFSI) complex with a Li_0.5_NiO_2_ cathode surface. (E) Differential scanning calorimetry thermograms of delithiated (to 4.2 V) NCM811 cathode materials. (F) High-angle annular dark-field scanning transmission electron microscopy images of NCM811 cathode particles (after 150 cycles in 1 M LiTFSI-DOL/DME [left] and 4 M LiFSI-PC/FEC [right]). (G) XPS F1s spectra of NCM811 cathode materials (after 150 cycles in 1 M LiTFSI-DOL/DME [left] and 4 M LiFSI-PC/FEC [right]).

A prerequisite condition for battery electrolytes is to ensure electrochemical stability with the battery components exposed to the electrolyte. The oxidation/reduction stability of the 4 M LiFSI-PC/FEC electrolyte was examined using LSV analysis. As a control sample, 1 M LiTFSI in DOL/ DME (= 1/1 v/v) (denoted as 1 M LiTFSI-DOL/DME) was chosen. 1 M LiTFSI-DOL/DME, which is known as a representative ether-based electrolyte, has been widely investigated with Li metal anodes (Yang et al., 2017, Zhang et al., 2017). However, its poor oxidation stability prohibits its use with high-voltage cathodes. In contrast to 1 M LiTFSI-DOL/DME, which is electrochemically unstable above ∼4.0 V (versus Li/Li^+^), the 4 M LiFSI-PC/FEC electrolyte has a far superior oxidation stability (Figure 3A). This, in concert with its above noted reduction stability with Li metal, suggests its potential for utilization in full cells with high-voltage cathodes. This significant improvement in stability is attributed to the coordinated Li^+^-FSI^−^-solvent clusters in the 4 M LiFSI-PC/FEC electrolyte. In addition, the ionic conductivity of this 4 M LiFSI-PC/FEC electrolyte over a wide temperature range is quite high (Figure S9).

The influence of the electrolytes on the charge/discharge cyclability and cathode electrolyte interface (CEI) of NCM811 cathodes was examined using a capacity-matched Li metal-NCM811 full-cell (NCM811) cathode (areal capacity = 3.0 mAh cm^−2^) and Li metal anode (3.0 mAh cm^−2^), in which the Li metal anode was fabricated by electrochemical deposition of Li on a copper (Cu) current collector in a separate cell (using a 4 M LiFSI-PC/FEC electrolyte). The full cell was cycled at a charge/discharge current density of 0.35/0.7 mA cm^−2^ over a voltage range of 3.0–4.2 V (versus Li/Li^+^). Notably, the cell with the 4 M LiFSI-PC/FEC electrolyte displayed exceptional cycling performance (capacity retention ∼92% after 150 cycles) (Figures 3B and S10), in marked contrast to control cells having different Li salt concentrations (1 M and 5 M LiFSI-PC/FEC) as well as 1 M LiTFSI-DOL/DME that showed poor cyclability and large cell polarization. This result was verified by examining the EIS spectra (Figure S11). The cell resistance of 1 M LiTFSI-DOL/DME was substantially increased after the cycle test due to the continuous electrolyte decomposition. In contrast, the 4 M LiFSI-PC/FEC showed stable cell resistance during the cycle test. To further elucidate why this exceptional cyclability of the full cell was achievable, the CEI layers on the cycled NCM811 cathodes were analyzed. A thin CEI layer (∼9 nm) was formed after 150 cycles when using 4 M LiFSI-PC/FEC (Figure 3C), whereas the 1 M LiTFSI-DOL/DME electrolyte generated a thick CEI layer (∼40 nm) (Figure S12) due to oxidative decomposition of the DOL and DME molecules (Xu, 2004). Moreover, the NCM811 particles remained stable in the 4 M LiFSI-PC/FEC electrolyte, whereas the 1 M LiTFSI-DOL/DME electrolyte caused extensive microcracks to form inside the NCM811 particles (Figure S13). This result demonstrates that the 4 M LiFSI-PC/FEC electrolyte, due to the highly coordinated structure of the Li^+^-FSI^−^-solvent clusters, suppresses H-transfer reactions of the solvents on the NCM811 particles and enables the formation of a stable/robust CEI layer on the cathode active particles.

The reactivity of PC, PC(LiFSI), FEC, and FEC(LiFSI) complexes was investigated by DFT calculations using the LiNiO_2_ and Li_0.5_NiO_2_ model cathode surfaces as discussed in detail in the Supplemental Information. All these complexes were found to be stable on the fully discharged LiNiO_2_ cathode surface with the PC(LiFSI) and FEC(LiFSI) complexes being the most stable (Figures 3D, S14, and S15). However, FEC and especially PC were found to undergo H-transfer to the oxygen of the partially charged cathode surface Li_0.5_NiO_2_ (Figures S16–S18), even when all the PC molecules are coordinated to LiFSI, as expected in the highly concentrated electrolyte. The FEC⋅_-H_ radical, produced as a result of H-transfer to cathode surface, undergoes condensation reaction and forms a partially fluorinated oligomeric CEI that is expected to further polymerize after further H-transfer to the cathode, eventually forming a highly fluorinated, cross-linked, and electrochemically stable polymer CEI (Figure S19). The FSI_-F_ species likely generated at lower potentials on NCM, which were noted earlier, may also function as radical scavengers near the interface at higher potentials. The overall positive effects of concentrated electrolytes observed here further bolsters the case for their use in promoting the stability of carbonate electrolytes in batteries featuring Ni-rich cathode materials.

It is known that upon charging process (*i.e.*, delithiation from cathode), oxygen defect formation and release from NCM-based cathode occur (Kong et al., 2019), thereby accelerating interfacial side reactions (including exothermic reactions) between the cathode active material and electrolyte components. The interfacial exothermic reaction of delithiated NCM811 with 4 M LiFSI-PC/FEC (and 1 M LiTFSI-DOL/DME) was therefore examined by differential scanning calorimetry (Figure 3E). The sample of delithiated NCM811 combined with 1 M LiTFSI-DOL/DME displayed a large exothermic heat release (ΔH = 783.6 J g^−1^) and low exothermic peak temperature (T_peak_ = 220.2°C), indicating that vigorous interfacial exothermic reactions occurred. By comparison, the exothermic heat release was substantially reduced (ΔH = 326.7 J g^−1^) and the exothermic peak temperature was shifted to a higher temperature (T_peak_ = 250.9°C) when the delithiated NCM811 was combined with 4 M LiFSI-PC/FEC instead. This result indicates that 4 M LiFSI-PC/FEC effectively suppresses the interfacial exothermic reactions that typically occur with NCM811 cathode material.

The effect of the electrolytes on structural changes of the NCM811 active material was evaluated, with a focus on oxygen release and the accompanied phase transformation. After 150 cycles, a characteristic XPS O1s peak at 530.8 eV, which is assigned to metal oxides, was observed at 5 nm depth from the NCM811 surface in contact with 1 M LiTFSI-DOL/DME (Figure S20A). In contrast, the O1s peak was hardly detected when 4 M LiFSI-PC/FEC was instead used (Figure S20B) due to the above-noted suppression of oxygen release from NCM811. The oxygen release accelerates the migration of transition metal ions through adjacent tetrahedral sites (Genevois et al., 2014, Kim et al., 2016). The resulting phase transformation of NCM811 was investigated using high-angle annular dark-field scanning transmission electron microscopy. Pristine NCM811 particles have a well-defined layered *R(−)3m* phase (Figure S21). It is known that delithiated NCM811 is vigorously reactive and thus easily transformed into an inactive rock salt *Fm(−)3m* phase (Jung et al., 2014), resulting in unwanted capacity fading. For the 1 M LiTFSI-DOL/DME electrolyte, a considerable proportion of the NCM811 surface was transformed into cation-mixed layers (>10 nm) with a rock salt-like structure (Figure 3F, left), but a very different behavior was observed when using 4 M LiFSI-PC/FEC. For the latter, the phase transformation occurs only to a limited depth (<3 nm) from the NCM811 surface (Figure 3F, right), presumably due to the advantageous effect of the thin CEI layer on the structural stability of NCM811.

The phase transformation of NCM811 noted above tends to cause transition metal dissolution. A characteristic XPS F1s peak at 684.7 eV, corresponding to LiF/MnF_x_ by-products that are known to arise from undesired interfacial side reactions between electrolytes and cathode materials (Jiao et al., 2018a, Jiao et al., 2018b), was significantly reduced in magnitude for the 4 M LiFSI-PC/FEC electrolyte relative to that of 1 M LiTFSI-DOL/DME (Figure 3G). This result was verified by a time-of-flight secondary ion mass spectroscopy analysis of MnF_2_. It is clear that the formation of the LiF/MnF_2_ by-products is significantly suppressed when using 4 M LiFSI-PC/FEC (Figure S22).

These results demonstrate that the 4 M LiFSI-PC/FEC electrolyte enables the formation of thin and stable CEI layers on NCM811, thereby greatly improving the structural stability (specifically, mitigation of oxygen release and the resulting phase transformation) of the NCM811 cathode material. The aforementioned CEI layers, in collaboration with the stabilized SEI layers on Li metal anodes (described in Figure 2), played a viable role in the superior electrochemical performance during the full cell operation.

### Ultra-high-Energy-Density/Safer Li Metal Full Cells Enabled by Highly Coordinated Nonflammable Carbonate Electrolytes

A few publications have recently reported the use of thin Li metal anodes to develop practically meaningful high energy-density Li metal full cells (Kim et al., 2018, Niu et al., 2019). However, combining thin Li metal anodes with high-capacity/high-voltage cathodes has remained a challenging task. Moreover, safety concerns of the resulting Li metal full cells have not been fully resolved.

**Figure 4:**
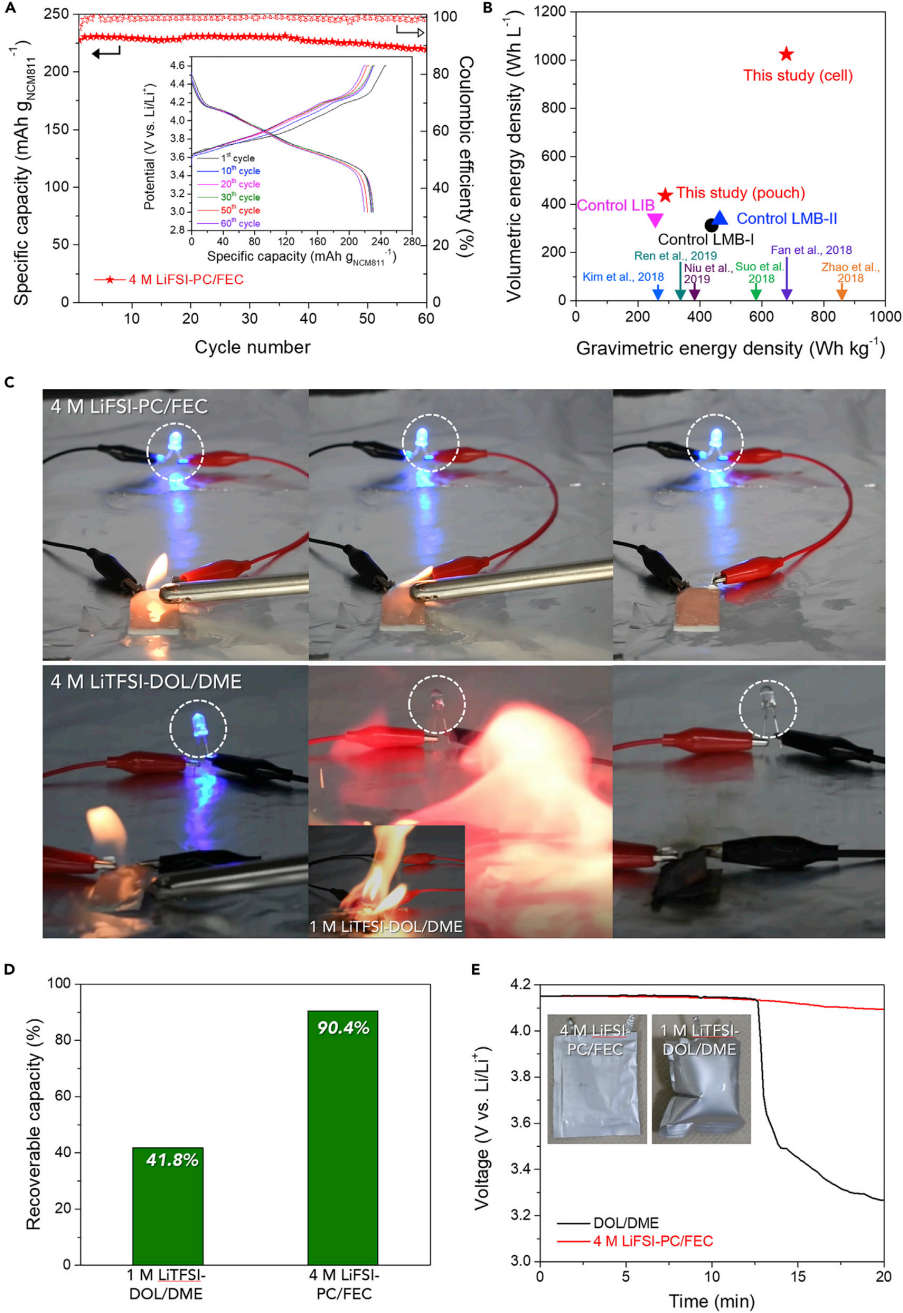
Ultra-high-Energy-Density Li Metal Full Cells (A) Cycling performance of Li (4 mAh cm^−2^)||NCM811 (4.8 mAh cm^−2^) cells over a voltage range of 3.0–4.6 V (inset shows the charge/discharge profiles). (B) Comparison of gravimetric/volumetric energy densities between this study and previously reported LMBs and control samples. Detailed information on the cell weight/volume of each system is provided in Table S1. (C–E) Safety analysis of Li metal full cells in various abuse conditions: (C) Combustion test of 10 mAh pouch-type cells charged to 4.2 V: 4 M LiFSI-PC FEC (upper) and 4 M LiTFSI-DOL/DME (bottom, inset shows the result of 1 M LiTFSI-DOL/DME). (D) Capacity retention of 10 mAh pouch-type cells charged to 4.2 V after the high-temperature storage test (60°C/24 h). (E) Hot-box test (130°C) of 500 mAh pouch-type cells charged to 4.2 V (inset shows the photographs of the cells after the hot-box test).

We fabricated a high-energy-density Li metal full cell by assembling a high-capacity NCM811 cathode (4.8 mAh cm^−2^ and ∼65 μm, shown in Figure S23A) and a low-capacity Li metal anode (4.0 mAh cm^−2^ and ∼35 μm, shown in Figure S23B) that was fabricated by electrochemical deposition on a Cu current collector, in which the capacity excess of the Li metal over the NCM811 cathode was 0.83. Note that the Li metal full cell was cycled over a voltage range of 3.0–4.6 V. The high cutoff charge voltage often gives rise to oxidative decomposition of conventional electrolytes. Under these harsh operating conditions (*i.e.*, 4.6 V charge cutoff voltage and thin Li metal anode with low areal capacity ratio of anode/cathode [<1.0]), the Li metal full cell displayed excellent cycling performance without noticeable polarization over 60 cycles (Figure 4A). Moreover, the cycled NCM811 particle maintained its spherical shape in the 4 M LiFSI-PC/FEC electrolyte, although some microcracks were formed, whereas severe structure disruption was observed in the 1 M LiTFSI-DOL/DME electrolyte along with the particle disintegration (Figure S24).

The gravimetric/volumetric energy densities of the Li metal full cell were compared with those of previously reported LMBs (Figure 4B and Table S1). Notably, the Li metal full cell reported in this study exhibited ultra-high energy densities (679 Wh kg_cell_^−1^/1,024 Wh L_cell_^−1^ and 288 Wh kg_pouch_^−1^/437 Wh L_pouch_^−1^) that far exceed those attainable with previous cell chemistries (for which the cell- and pouch-based energy densities were estimated on the basis of the total weight and volume of the Li metal anode [excluding a Cu current collector], NCM811 cathode [excluding an Al current collector], and separator, and the total weight and volume of all cell components [including the electrodes, current collectors, separators, electrolytes, packaging substances, and sealant taps; Table S2], respectively). To further investigate the effect of cell configuration on the energy density, control cells including an LIB (*i.e.*, composed of NCM811 cathode [3.0 mAh cm^−2^]||graphite anode [3.3 mAh cm^−2^]) and LMBs (*i.e.*, control LMB-I = NCM811 cathode [3.8 mAh cm^−2^]||thick Li metal anode [200 μm] (Markevich et al., 2017) and control LMB-II = NCM811 cathode [1.2 mAh cm^−2^]||thin Li metal anode [20 μm] (Liu et al., 2018a, Liu et al., 2018b, Suo et al., 2018)) were fabricated. Despite the use of the same electrolyte (4 M LiFSI-PC/FEC), all control cells failed to reach comparable high energy densities (Figure S25). This comparative study demonstrates that tailoring of the full cell configuration, which simultaneously fulfills the requirements of both the cathode (high-capacity/high-voltage) and anode (low-capacity thin Li metal), is essentially needed for the development of (gravimetric/volumetric) high-energy-density LMBs.

In addition to the aforementioned electrochemical performance, efforts should be made to ensure that Li metal full cells operate safely. The solvents (*i.e.*, PC and FEC) of 4 M LiFSI-PC/FEC are known to have high thermal stability and even nonflammability (Shi et al., 2017) and are thus expected to add beneficial effects to the cell safety. The isothermal thermogravimetric analysis curves at 80°C (Figure S26), which is known as a critical temperature for provoking the spontaneous thermal runaway of cells, clearly indicated the superior thermal stability (*i.e.*, reduced volatility) of 4 M LiFSI-PC/FEC (weight loss after 90 min = 5.0 wt. %) compared with 1 M LiTFSI-DOL/DME (= 64.7 wt. %). Furthermore, it is generally believed that concentrated electrolytes are thermally stable due to strong ion-solvent interactions. As a representative example of concentrated electrolytes (Jiao et al., 2018b), 4 M LiTFSI-DOL/DME was prepared and its volatility was measured at 80°C. The weight loss after 90 min was found to be 31.2 wt. % (Figure S26). Although this value was improved relative to the result for 1 M LiTFSI-DOL/DME, it is still much larger than that of 4 M LiFSI-PC/FEC, thus evincing the importance of solvent characteristics in the thermal properties of electrolytes.

The safety tolerance of Li metal full cells was then evaluated in various abuse conditions. 10 mAh pouch-type cells (composed of Li metal (∼4 mAh cm^−2^ and 20 μm) anode and NCM811 cathode [4 mAh cm^−2^]) were charged to 4.2 V and then exposed to a flame, after removal of their packaging. Interestingly, the cell containing 4 M LiFSI-PC/FEC operated a light-emitting diode (LED) lamp even when exposed to the flame (Figure 4C, top, and Video S1), whereas the cells with 4 M LiTFSI-DOL/DME (and 1 M LiTFSI-DOL/DME) instantly caught fire (Figure 4C, bottom, and Videos S2 and S3). Such an exceptional improvement in the cell safety is due to the use of nonflammable 4 M LiFSI-PC/FEC (instead of highly flammable ether solvents). This substantial safety improvement was further verified by examining the nonflammability of the cell components. The Li metal, NCM811 cathode, and even polyethylene separator, all of which were pre-soaked with 4 M LiFSI-PC/FEC, were not ignited upon exposure to a flame (Figure S27), which was not the case for the electrolytes with ether solvents. In addition, the capacity retention of the 4.2 V-charged pouch-type cells after exposure to 60°C for 24 h was estimated. The cell containing 4 M LiFSI-PC/FEC had a much higher capacity retention (90.4%) than that with 1 M LiTFSI-DOL/DME (41.8%) (Figure 4D). This improvement in the high-temperature storage test became more pronounced at a higher temperature of 80°C (Figure S28). To further explore the safety behavior of the Li metal full cell, we conducted a hot-box test with 500 mAh pouch-type cells. The voltage of the 4.2 V-charged cells was monitored as a function of elapsed time at 130°C. In contrast to the result of 1 M LiTFSI-DOL/DME, the cell with 4 M LiFSI-PC/FEC maintained its voltage above 4 V without dimensional swelling or distortion (Figure 4E).

Video S1. Combustion Test of 10-mAh Pouch-type Cell with 4 M LiTFSI-PC/FEC, Related to Figure 4C

Video S2. Combustion Test of 10-mAh Pouch-type Cell with 4 M LiTFSI-DOL/DME, Related to Figure 4C

Video S3. Combustion test of 10-mAh Pouch-type Cell with 1 M LiTFSI-DOL/DME, Related to Figure 4C

In summary, we have developed the ultra-high-energy-density and nonflammable Li metal full cells by coupling low-capacity/thin Li metal anodes (4.0 mAh cm^−2^/35 μm) with high-capacity/high-voltage NCM811 cathodes (4.8 mAh cm^−2^/4.6 V) utilizing a 4 M LiFSI-PC/FEC electrolyte. This electrolyte has a unique coordination structure of Li^+^-FSI^−^-solvent clusters, which contributes to the formation both stable SEI and CEI layers. The resulting SEI and CEI layers enabled highly stable Li plating/stripping on the Li metal anode and maintained the structural stability of the NCM811 cathode particles, respectively. With these advantageous effects, the Li metal full cell achieved remarkably high energy densities (679 Wh kg_cell_^−1^/1,024 Wh L_cell_^−1^ and 288 Wh kg_pouch_^−1^/437 Wh L_pouch_^−1^) with stable performance under highly demanding cell configuration and operating conditions, while also exhibiting exceptional safety (nonflammability and normal cell operation even upon exposure to a flame). The coordinated nonflammable carbonate electrolyte strategy of this study, in combination with the rationally engineered cell configuration, is a tremendous step forward toward practical Li metal full cells.

## Methods

All methods can be found in the accompanying Transparent Methods supplemental file.

## Supplemental Information

Document S1. Transparent Methods, Figures S1–S28, and Tables S1–S3

## Footnotes

Supplemental Information can be found online at https://doi.org/10.1016/j.isci.2020.100844.
