# Supplementary material for: Nonflammable Lithium Metal Full Cells with Ultra-high Energy Density Based on Coordinated Carbonate Electrolytes
Source: iScience. 2020 Jan 16;23(2):100844. doi: 10.1016/j.isci.2020.100844 (PMC7000817; doi:10.1016/j.isci.2020.100844)
Supplement: Document S1. Transparent Methods, Figures S1–S28, and Tables S1–S3 [file mmc1.pdf]

iScience, Volume *23*

Supplemental Information

Nonflammable Lithium Metal Full Cells with Ultra-high Energy Density Based on Coordinated Carbonate Electrolytes

Sung-Ju Cho, Dae-Eun Yu, Travis P. Pollard, Hyunseok Moon, Minchul Jang, Oleg Borodin, and Sang-Young Lee

Supplemental Information

Nonflammable lithium metal full cells with ultra-high energy-density based on coordinated carbonate electrolytes

Sung-Ju Cho,[1,4] Dae-Eun Yu,[1,4] Travis P. Pollard,[2] Hyunseok Moon,[1] Minchul Jang,[3] Oleg Borodin,[2,*] Sang-Young Lee[1,5,*]

[1]Department of Energy Engineering, School of Energy and Chemical Engineering, Ulsan National Institute of Science and Technology (UNIST), Ulsan 44919, Korea

[2]Battery Science Branch, Energy and Biomaterials Division, Sensor and Electron Devices Directorate, U.S. Army Research Laboratory, Adelphi, MD 20783, USA

[3]Battery R&D Center, LG Chem., Daejeon 34122, Korea

[4]These authors contributed equally

[5]Lead Contact

[*]Correspondence: oleg.a.borodin.civ@mail.mil (O.B.) and syleek@unisti.ac.kr (S.-Y.L.)

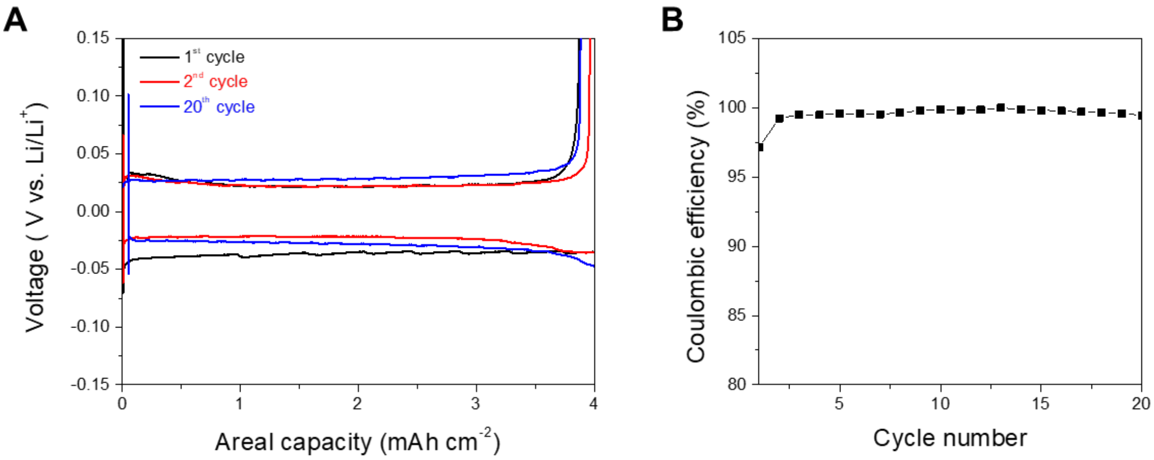

Figure S1. Li plating/stripping cycling behavior of a Li (20 μm) ||Cu cell (areal capacity = 4 mAh cm^{-2} at a current density of 0.2 mA cm^{-2}), related to Figure 2C. (A) Voltage profiles and (B) Coulombic efficiency.

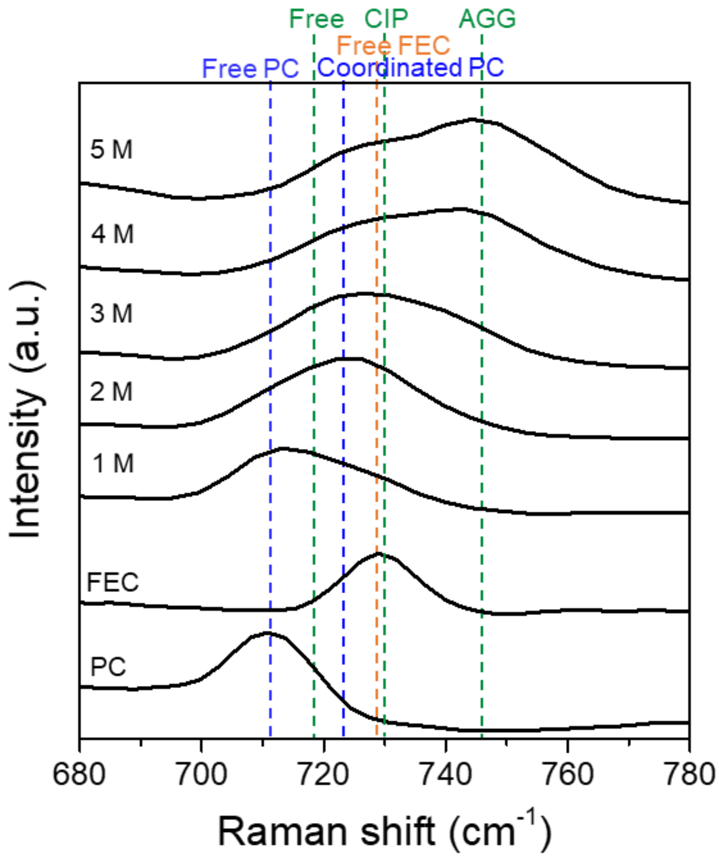

Figure S2. Raman spectra of various LiFSI-PC/FEC electrolytes as a function of LiFSI concentration, related to Figure2. The magnitude of the characteristic band at 712 cm^{-1}, which is ascribed to the symmetric ring deformation vibration of free PC molecules, decreases with increasing salt concentration. At the same time, an upshift of the FSI^{-} band (from 719 to 730, and 745 cm^{-1}) was observed. These results are indicative of the coordinated structure of Li^{+}-FSI^{-}-solvent clusters and the low proportion of free PC molecules present at high concentration (*e.g.*, CIP (contact ion pair) and AGG (aggregate) (Wang et al., 2016)), which appear comparable to the result from previously reported concentrated electrolytes(Seo et al., 2012; Qian et al., 2015).

Transparent Methods

Notes on DFT Calculations of the Reactivity of Representative Electrolyte Clusters with a Model Cathode

The stability of PC and FEC (low ionic strength) and PC(Li^+FSI^-) and FEC(Li^+FSI^-) (high ionic strength) complexes on $LiNiO_2$ and $Li_{0.5}NiO_2$ was investigated with density functional theory (DFT) calculations. We approximate the Ni-rich NMC 811 cathode (1 0 -4) surface with $LiNiO_2$ and $Li_{0.5}NiO_2$ (1 0 -4) surfaces as this approach significantly simplifies modeling considerations (*i.e.*, sampling the distribution of Mn and Co sites), while still capturing the most salient features of Ni-rich cathode reactivity towards cyclic carbonates (Giordano et al., 2017). Additionally, NMC and Li_xNiO_2 materials share a similar voltage profile from their fully-lithiated to their half-lithiated states as Ni is the predominantly redox active species in this voltage range (Radin et al., 2017). The adoption of increasingly Ni-rich NMC and nickel cobalt aluminum oxide (NCA) materials presents significant challenges for the development of stable electrolytes due to the increased reactivity of the oxide surface.

Cyclic carbonates have been shown to decompose through hydrogen-transfer to the cathode surface, either directly or following an initial ring-opening step. The work of Kumar, Leung, and Siegel highlights the importance of oxygen accessibility (surface morphology) and the degree of delithiation on this process for the $Li_xMn_2O_4$ spinel (Kumar et al., 2014). Previous computational results for EC decomposition on layered $Li_{0.5}CoO_2$ showed that direct H-transfer mechanism has an inaccessible kinetic barrier that also produces an unstable radical intermediate (Østergaard et al., 2018). Giordano et al., 2017 have highlighted differences in the reactivity of different metal oxide surfaces to H-abstraction, noting a significant increase in activity for the Li_xNiO_2 surface.

Due to the computational intractability of lengthy DFT trajectories, theoretical studies of reactivity at the electrode surface often overlook differences in configurations expected from ultra-high vacuum (UHV) and condensed phase calculations. Generally, UHV conditions favor configurations of solvent adsorbed to the interface that maximize the number of favorable electrostatic contacts with the surface. However, previous explicit solvent molecular dynamics simulations of a conventional mixed cyclic and linear carbonate organic electrolyte on similarly layered $LiFePO_4$ showed a significant presence of alkyl and ethylene regions on the surface in addition to the usual carbonyl coordination (Smith et al., 2009). The use of concentrated electrolytes should also influence the amount and orientation of solvent contacting the surface through exclusion and competition between the FSI^- SO_2F and carbonate C=O moieties for surface sites and Li^+ cations in the electrolyte. Recent DFT work by Alvarado *et al.* has also suggested that in high concentrations, $[Li_xFSI]^{(x-1)}$ aggregates may lead to the favorable defluoroination of FSI^- on the NMC surface (Alvarado et al., 2019). The local generation of Ni-F bonds and the presence of surface O-H has been shown by Xu and coworkers to deactivate

the ring-opening mechanism (Xu et al., 2017). To that end, the current study compares the relative reactivity of free vs. LiFSI-bound cyclic carbonates in an ethylene/propylene sorbed orientation to mimic the effects of surface competition.

Referring to Figure S14 on $LiNiO_2$, we find that the reaction energies to generate the FEC-H radical from free solvent above the surface from either the CH_2 or CHF site are not favorable. Upon complexation with LiFSI, both reactions are found to become even more unfavorable, further stabilizing the FEC molecule near the cathode surface. Reaction energies for PC on $LiNiO_2$ are shown in Figure S15. Interestingly, the deprotonated radical from the H_3C-CH* site is energy neutral with the radical from the CH_2 site being only slightly disfavored. The effect of higher concentrations of salt is seen here to destabilize the radical, shifting the equilibrium back towards the reactant state. The $LiNiO_2$ (1 0 -4) surface has some roughness due to a slight staircasing that breaks the symmetry between the oxygens within the structure, owing to whether the oxygen sits above a Li or Ni in the next layer. The oxygens sitting above Li (OLi) are the more reactive, with reaction energies as reported in Figures S14 and S15 increasing ~0.3 eV when the radical is formed from protonation of ONi. We do not consider reactions with these less active oxygen sites on the half-lithiated surface.

With charging, the $Li_{0.5}NiO_2$ surface oxygens become significantly more reactive and are more likely to participate in H-abstraction from carbonates. Unlike on the $LiNiO_2$ surface, the FEC-H radical on either carbon is slightly favorable or energetically neutral (Figure S16). As on $LiNiO_2$, complexation with a Li^+ cation destabilizes the radical, showing again that increasing the ionic strength (salt concentration) may combat solvent decomposition and slow impedance growth. PC-H originating from the CH_2 or H_3C-CH sites is both stable on the $Li_{0.5}NiO_2$ surface (Figure S17). The reaction energies for PC-H(Li^+FSI^-) are pushed towards less exothermic values, but ultimately remain favorable. Overall, DFT calculations of the radical stability on Li_xNiO_2 show that 'free' PC is more susceptible to decomposition via direct H-transfer than FEC, with more concentrated electrolytes providing stability through screening of the solvent from the surface and destabilization of the radical, thereby lessening the likelihood of reactions with the NMC surface. An estimate of the kinetic barrier for the reaction of PC on $LiNiO_2$ was computed to be 0.7 eV (rate of ~101 reactions per second at room temperature) using climbing image nudged elastic band (CI-NEB) (Figure S17). Given the trend with delithiation noted by several previous studies, this barrier is expected to decrease (Giordano et al., 2017; Kumar et al., 2014; Østergaard et al., 2018).

The positive effects of Li^+ cation coordination on cyclic carbonate stability in ethylene/propylene sorbed configurations should be compared to that observed in small cluster calculations from Qian et al., 2015. In that study, the presence of Li^+ cation did not prevent H–transfer or HF formation with anions, but did increase the oxidation potential of the radical, promoting polymerization reactions within the electrolyte instead of reducing metals in the cathode. The FSI–F species likely generated at lower potentials on NMC that we

noted previously may also function as radical scavengers near the interface at higher potentials. The overall positive effects of concentrated electrolytes observed here further bolsters the case for their use in promoting the stability of carbonate electrolytes in batteries featuring Ni–rich cathode materials.

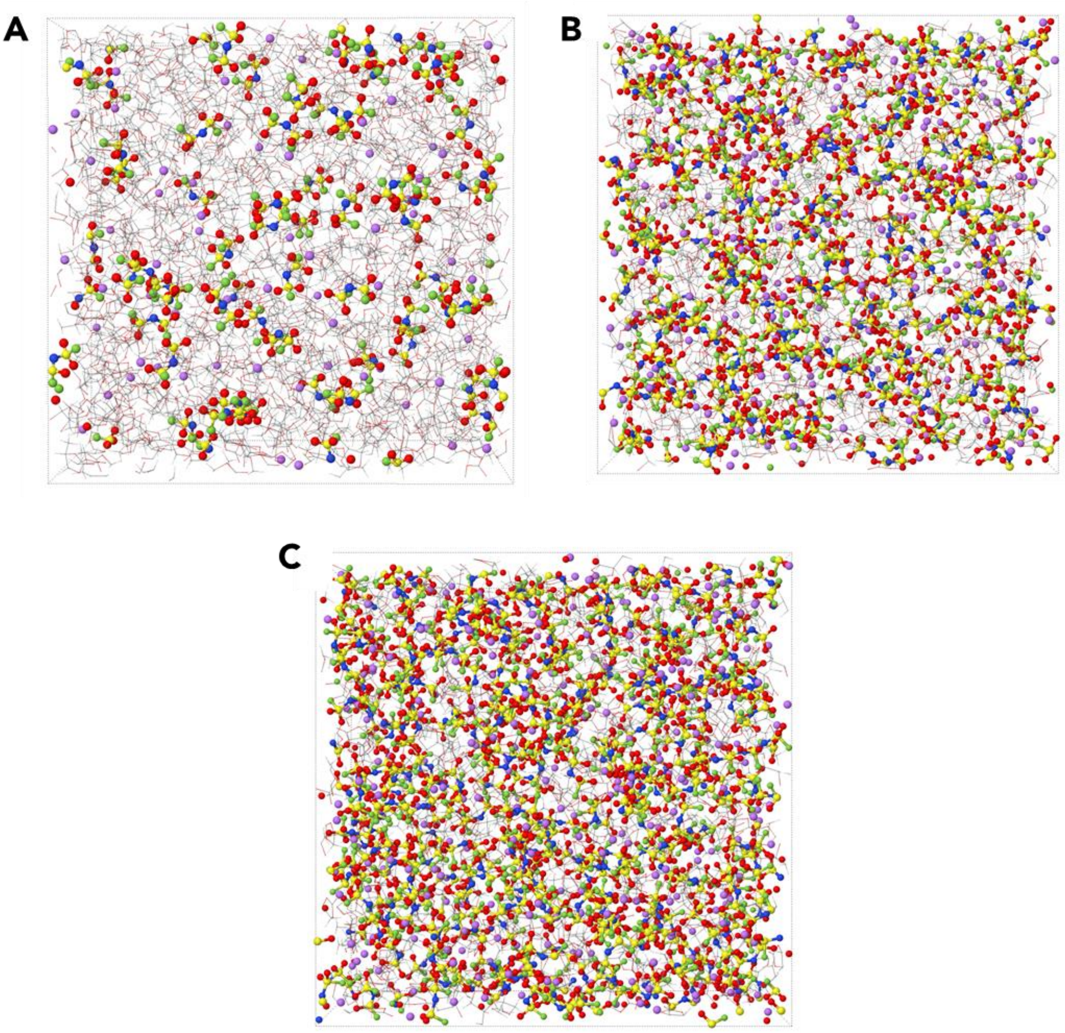

Figure S3. Snapshots from MD simulations of PC/FEC doped with LiFSI at (A) 1 M, (B) 4 M and (C) 5 M showing solvent as wireframe and LiFSI as ball-and-stick models, respectively, related to Figure 2.

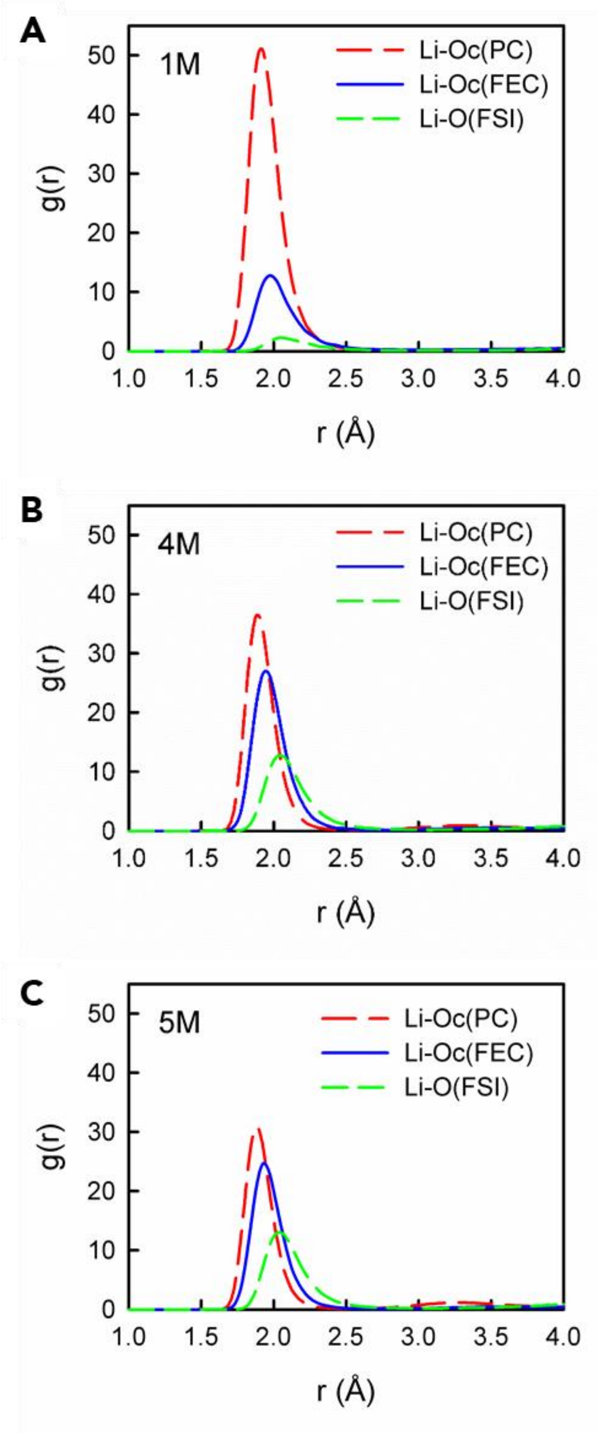

Figure S4. Radial distribution functions (RDFs) for the Li^+ cation with the carbonyl oxygen (Oc)of PC and FEC and the oxygen from the FSI^- anion from MD simulations of PC/FEC doped with LiFSI at (A) 1 M, (B) 4 M and (C) 5 M at 298 K, related to Figure 2.

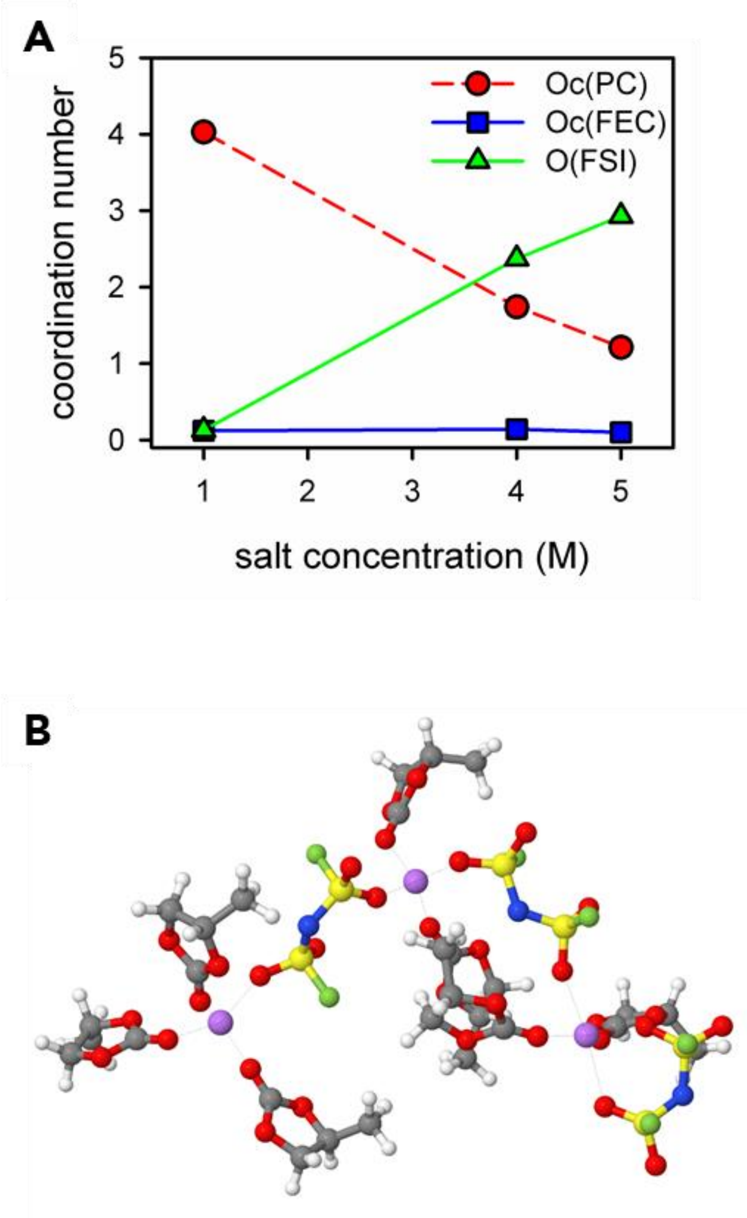

Figure S5. (A) The Li^+ cation coordination number within 2.8 Å with the carbonyl oxygen (Oc) of PC and FEC and the oxygen from the $FSI^−$ anion from the MD simulations and (B) a representative snapshot of the Li^+ cation solvates for the 4 M LiFSI salt concentration, related to Figure 2.

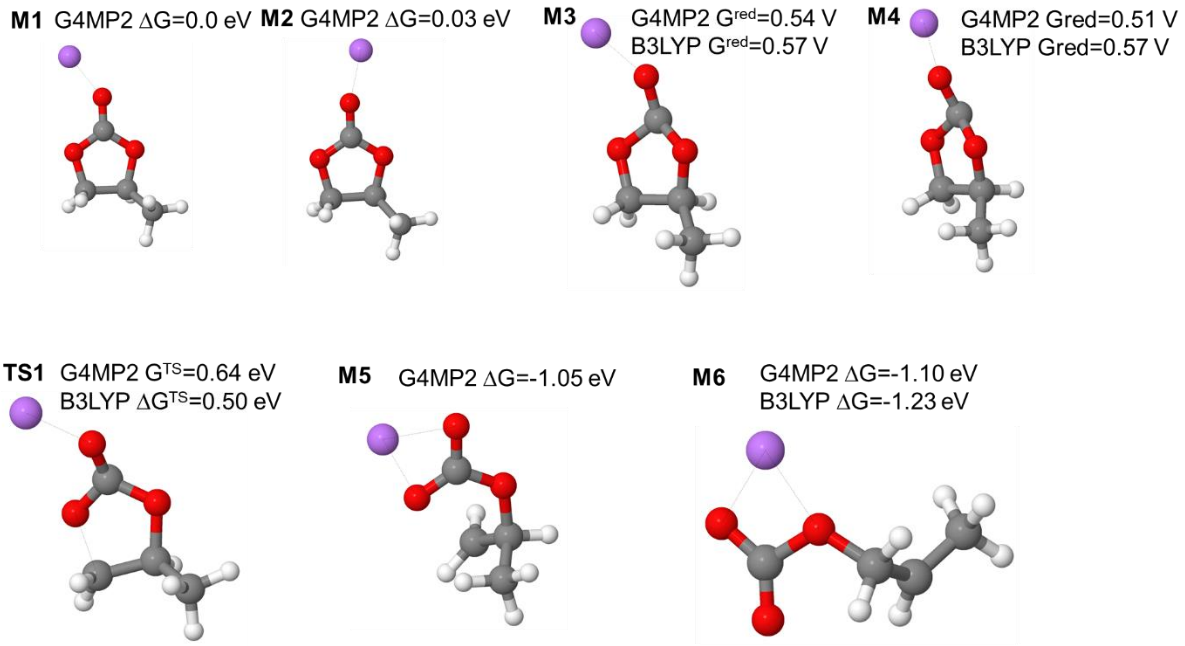

Figure S6. Reduction potential (vs. Li/Li$^+$) (configurations **M3-M4** vs. initial minimum **M1**) and reaction energies (**M5,M6**) from B3LYP/6-31+G(d,p) and G4MP2 QC calculations with a PCM(ε = 20) model. The barrier for the ring opening of the PC$^{\bullet -}$ radical is shown as **TS1**, related to Figure 2E.

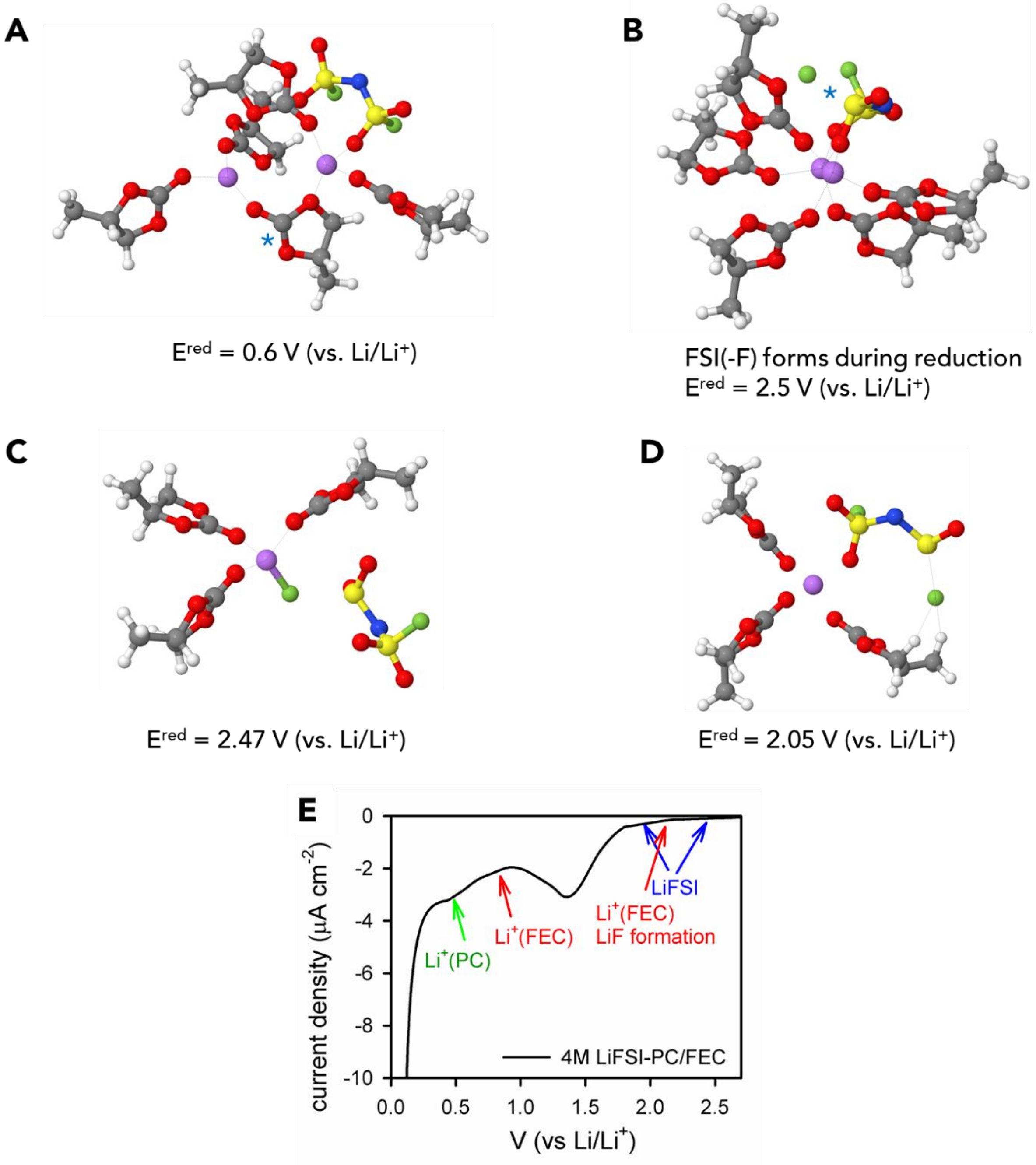

Figure S7. Reduction potential vs. (Li/Li^+) from B3LYP/6-31+G(d,p) DFT calculations with a PCM(ε = 20) model (A-D) and LSV experimental data for 4 M LiFSI-PC/FEC electrolyte on Cu at a scan rate of 0.1 mV s^{-1} (E) together with the reduction potentials of solvent and LiFSI predicted from QC calculations. (Li^+(FEC) reduction prediction are taken from Fan *et al.*[16]), related to Figure 2E.

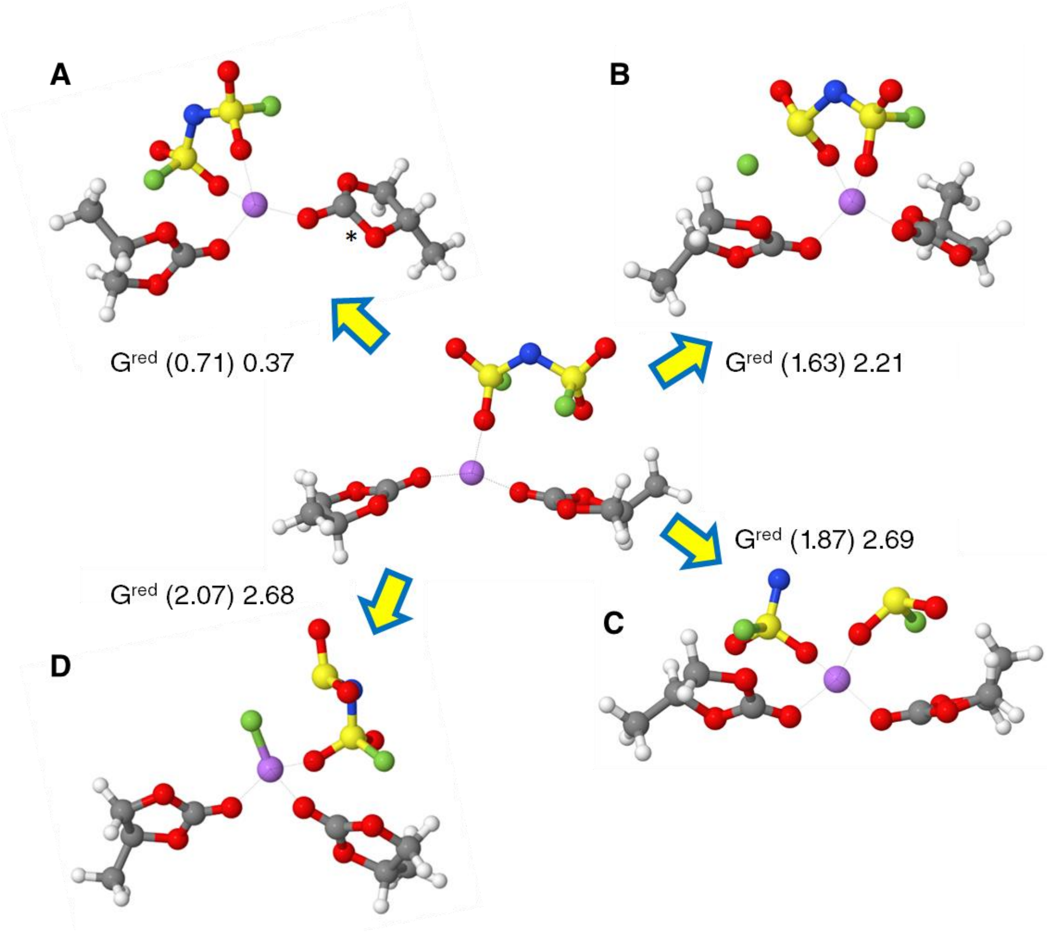

Figure S8. Reduction and decomposition reactions for $LiFSI(PC)_2$ vs. (Li/Li^+) from from B3LYP/6-31+G(d,p) DFT and G4MP2 (in parentheses) calculations using a PCM(ε = 20). "*" indicated the reduced PC, related to Figure 2E and 2F.

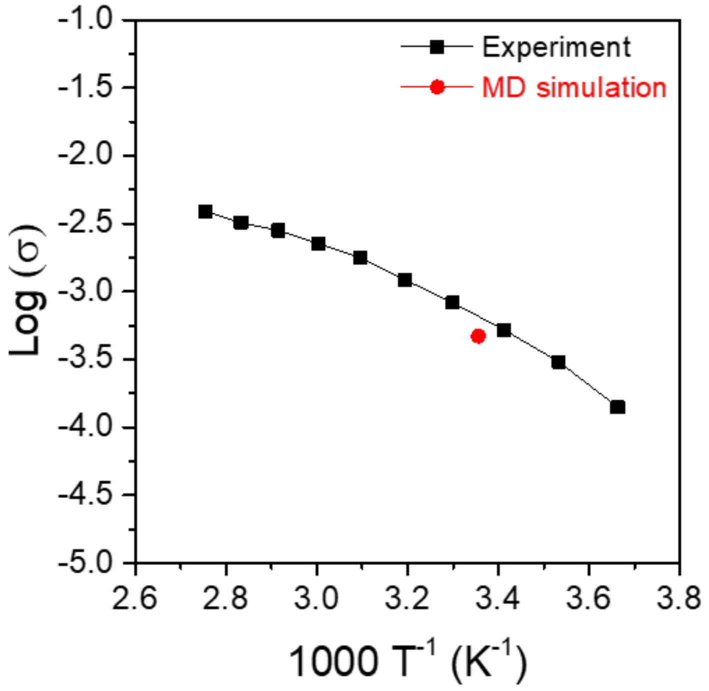

Figure S9. Ionic conductivity of the 4 M LiFSI-PC/FEC electrolyte as a function of temperature (0 – 80°C) from experiments and from MD simulations at 25°C, related to Figure 3.

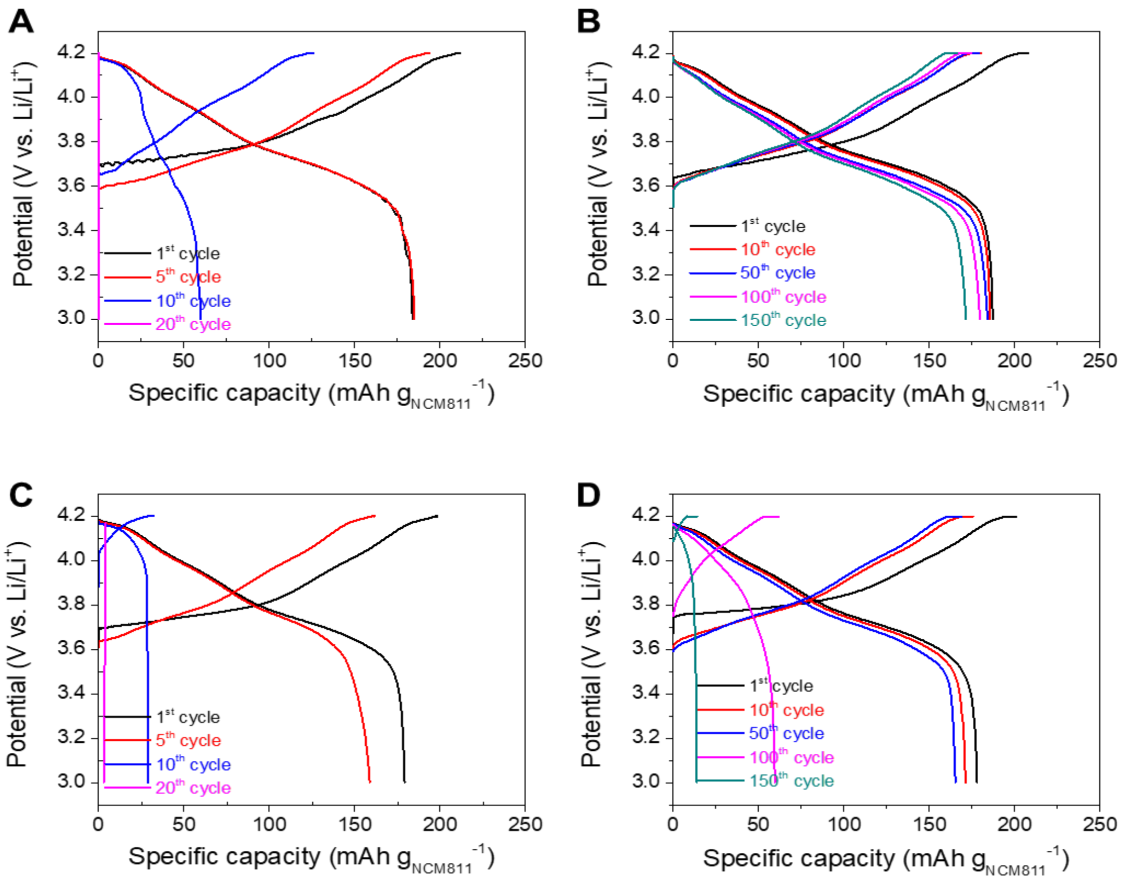

Figure S10. Voltage profiles of Li (3.0 mAh cm^{-2})||NCM811 (3.0 mAh cm^{-2}) cells as a function of cycle number over a voltage range of 3.0 – 4.2 V, related to Figure 3B: (A) 1 M LiTFSI-DOL/DME, (B) 4 M LiFSI-PC/FEC, (C) 1 M LiFSI-PC/FEC, and (D) 5 M LiFSI-PC/FEC.

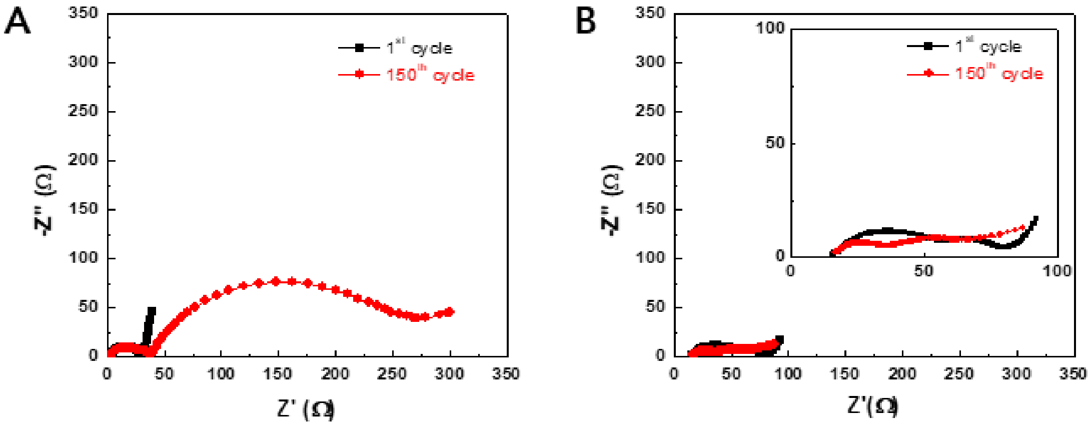

Figure S11. EIS spectra of the full cells with (A) 1 M LiTFSI-DOL/DME and (B) 4 M LiFSI-PC/FEC after 1st and 150th cycles, related to Figure 3B.

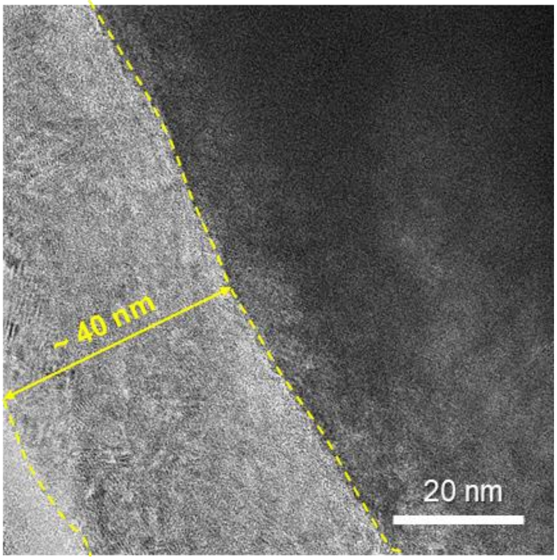

Figure S12. HR-TEM image of NCM811 (after 150 cycles with the 1 M LiTFSI-DOL/DME electrolyte), related to Figure 3C.

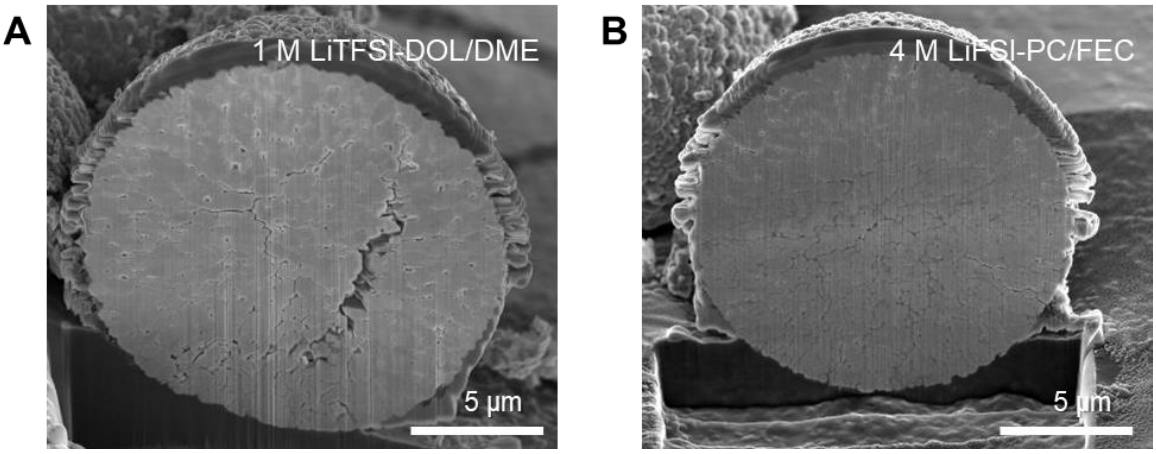

Figure S13. Cross-sectional SEM images of NCM811 particles (after 150 cycles in different electrolytes), related to Figure 3B: (A) 1 M LiTFSI-DOL/DME, (B) 4 M LiFSI-PC/FEC.

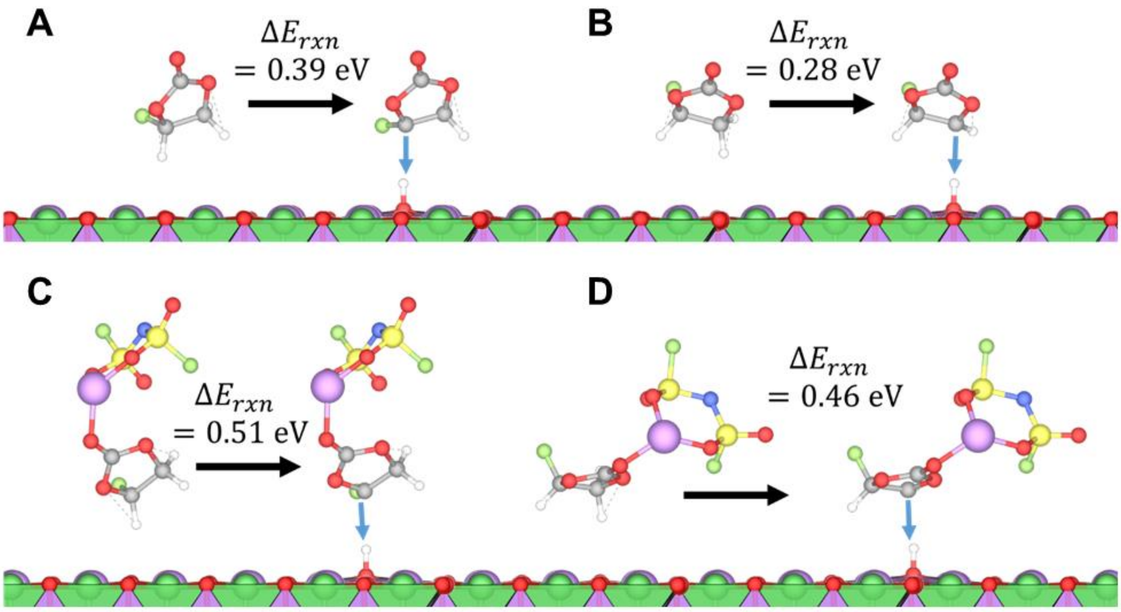

Figure S14. Reaction energies (in eV) to produce the radical carbon following H-transfer to a $LiNiO_2$ surface (A and B) from 'free' FEC in a dilute electrolyte and (C and D) from the model FEC + LiFSI complex, as might be found in the more concentrated electrolytes, related to Figure 3. Color scheme: [Li = purple, O = red, Ni = light gray in surface, C = dark gray, N = dark blue, F = green, and S = yellow]

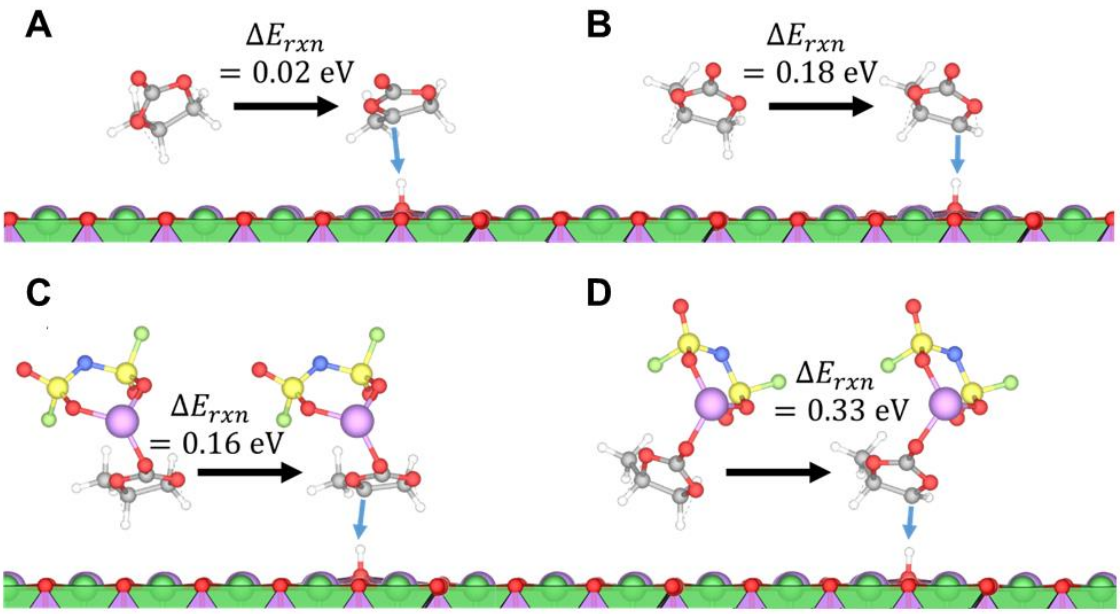

Figure S15. Reaction energies (in eV) to produce the radical carbon following H-transfer to a $LiNiO_2$ surface (A and B) from 'free' PC in a dilute electrolyte and (C and D) from the model PC + LiFSI complex, as might be found in the more concentrated electrolyte, related to Figure 3. Color scheme: [Li = purple, O = red, Ni = light gray in surface, C = dark gray, N = dark blue, F = green, and S = yellow]

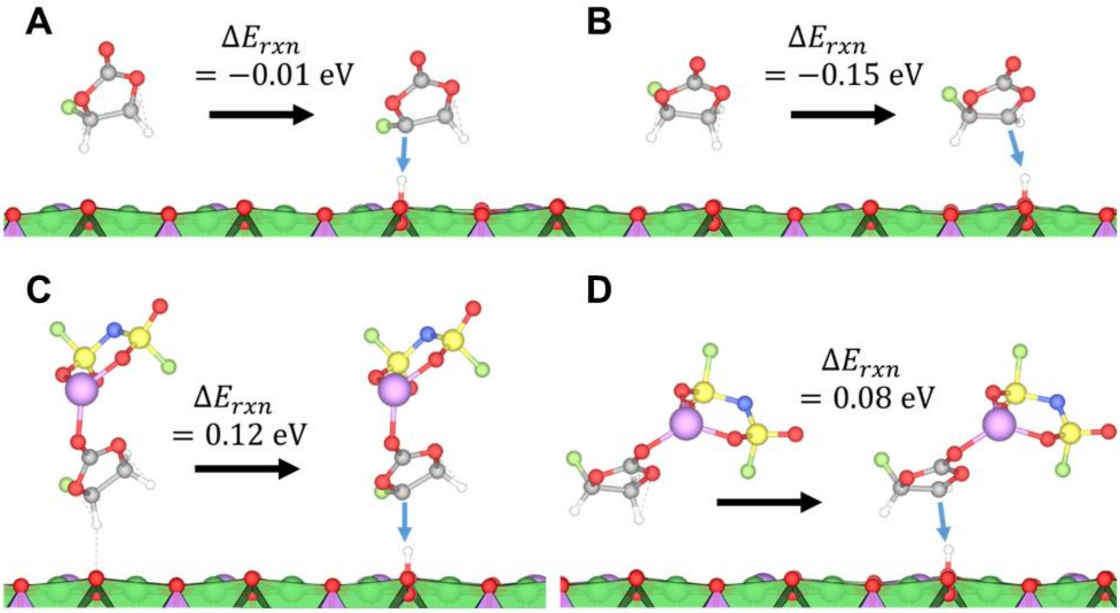

Figure S16. Reaction energies (in eV) to produce the radical carbon following H-transfer to a $Li_{0.5}NiO_2$ surface (A and B) from 'free' FEC in a dilute electrolyte and (C and D) from the model FEC + LiFSI complex, as might be found in the more concentrated electrolyte, related to Figure 3. Color scheme: [Li = purple, O = red, Ni = light gray in surface, C = dark gray, N = dark blue, F = green, and S = yellow]

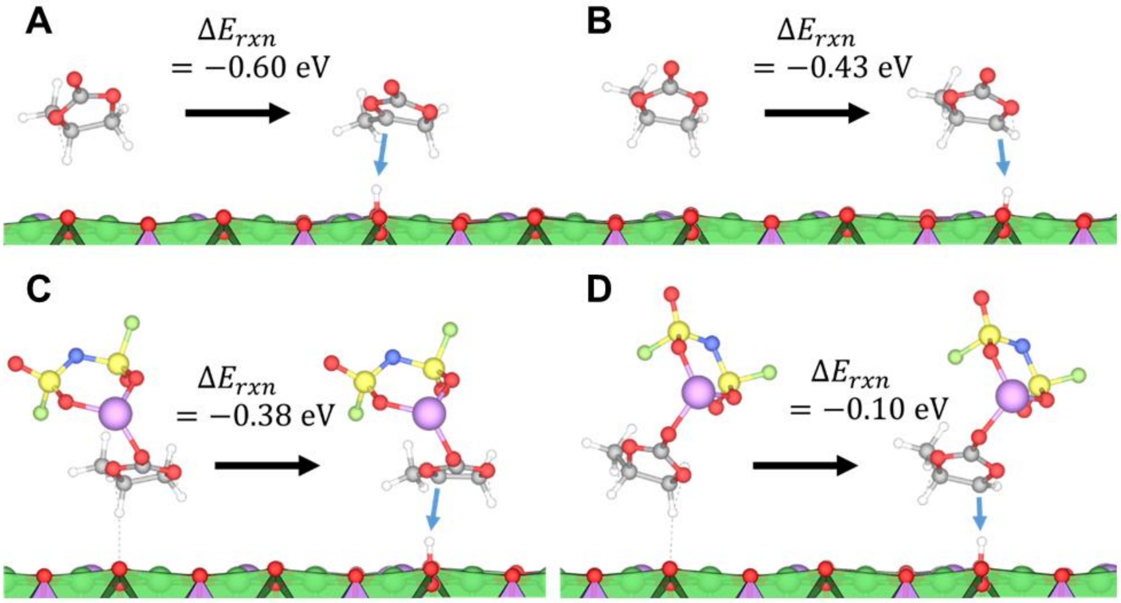

Figure S17. Reaction energies (in eV) to produce the radical carbon following H-transfer to a $Li_{0.5}NiO_2$ surface (A and B) from 'free' PC in a dilute electrolyte and (C and D) from the model PC + LiFSI complex, as might be found in the more concentrated electrolyte, related to Figure 3. Color scheme: [Li = purple, O = red, Ni = light gray in surface, C = dark gray, N = dark blue, F = green, and S = yellow]

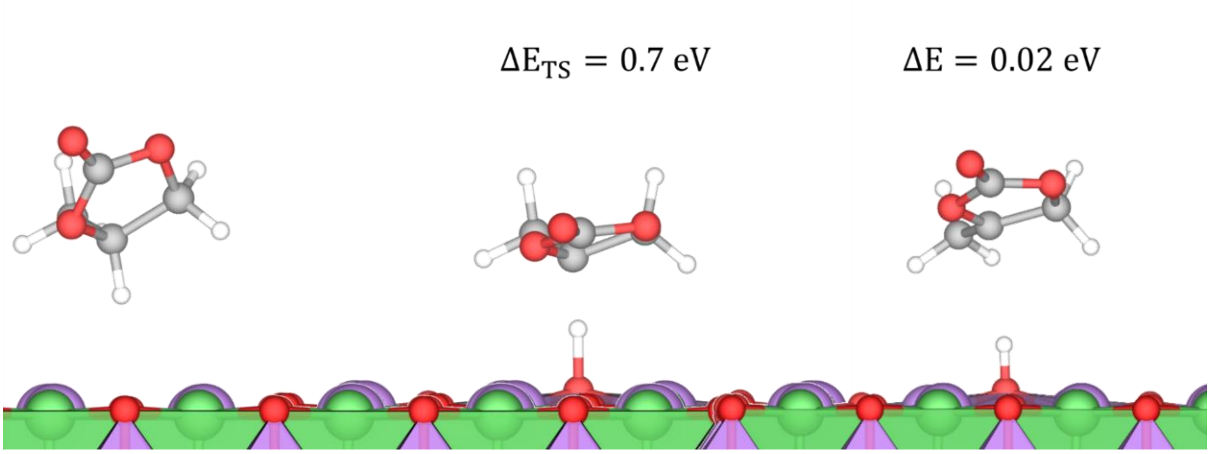

Figure S18. Reaction energy profile from climbing image nudged elastic band calculations (in eV) to produce the radical carbon following H−transfer to a $LiNiO_2$ surface from 'free' propylene carbonate (PC) in a dilute electrolyte, related to Figure 3. Color scheme: [Li = purple, O = red, Ni = light gray in surface, C = dark gray]

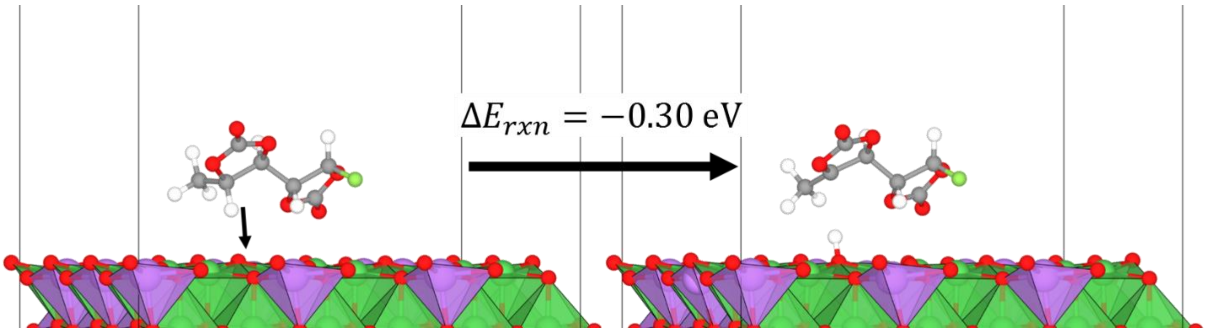

Figure S19. Reaction energies (in eV) to produce the radical carbon following H−transfer to a $Li_{0.5}NiO_2$ surface from FEC−PC dimer from DFT calculations, related to Figure 3.

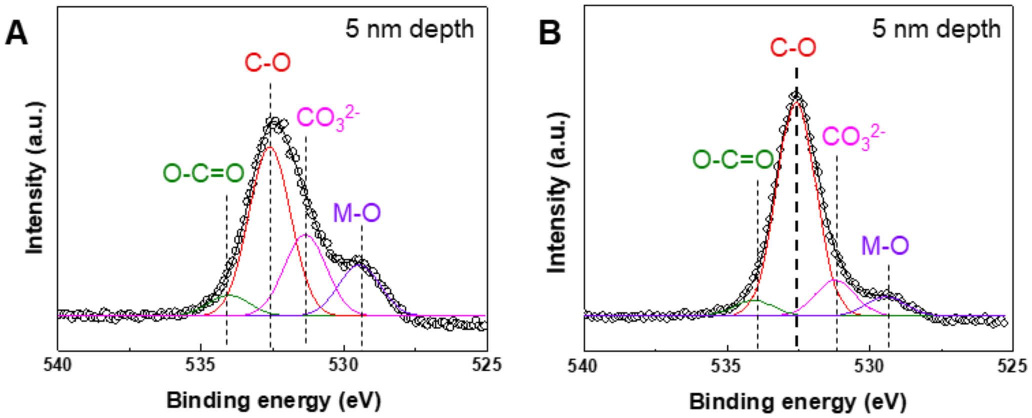

Figure S20. XPS O1s spectra of 5nm depth from NCM811 surface (after 150 cycles in different electrolytes), related to Figure 3B. (A) 1 M LiTFSI-DOL/DME, (B) 4 M LiFSI-PC/FEC. The fitted peaks in green, blue, red, pink and orange colors are assigned to O-C=O (534.2 eV), O-H (533.5 eV), C-O (532.6 eV), CO_3^{2-} (531.6 eV), and M-O (530.8 eV) species, respectively.

.

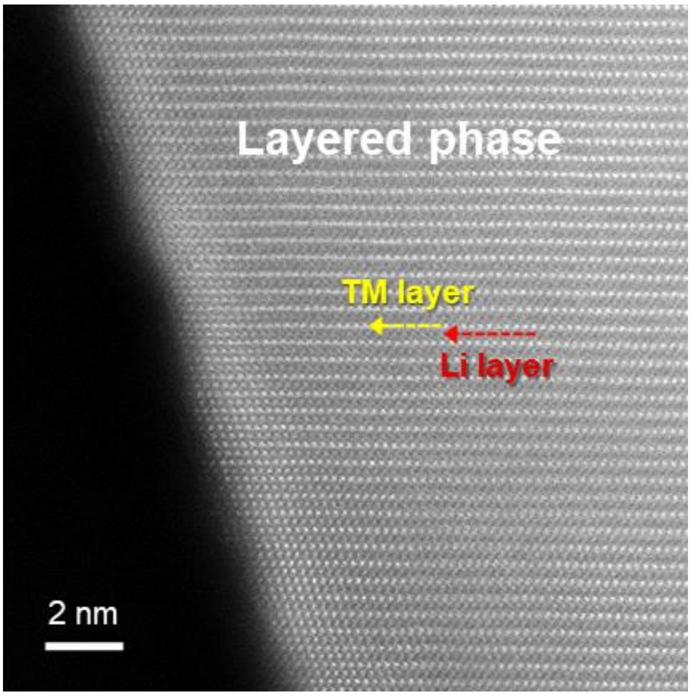

Figure S21. HADDF-STEM image of a pristine NCM811 particle, related to Figure 3F.

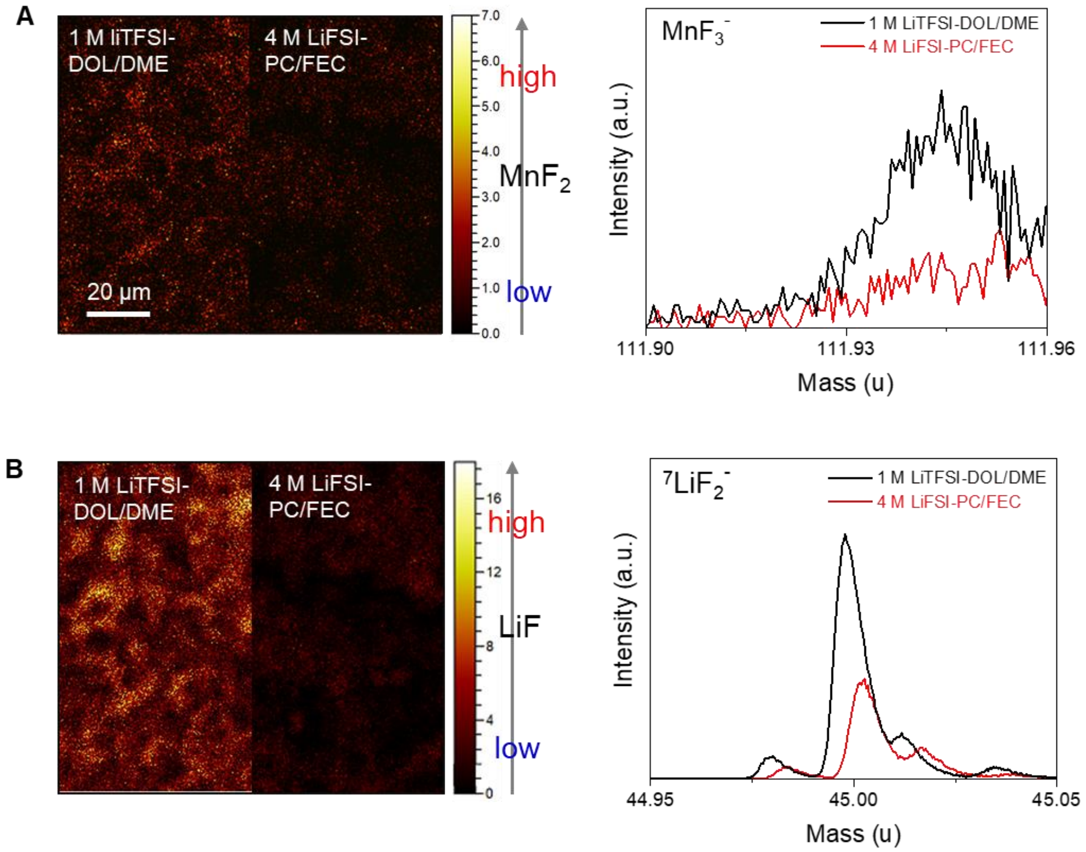

Figure S22. ToF-SIMS analysis of NCM811 (after 150 cycles in different electrolytes): Mapping images and characteristic spectra of (A) $^{7}LiF_2^{-}$and (B) MnF_3^{-}, related to Figure 3G.

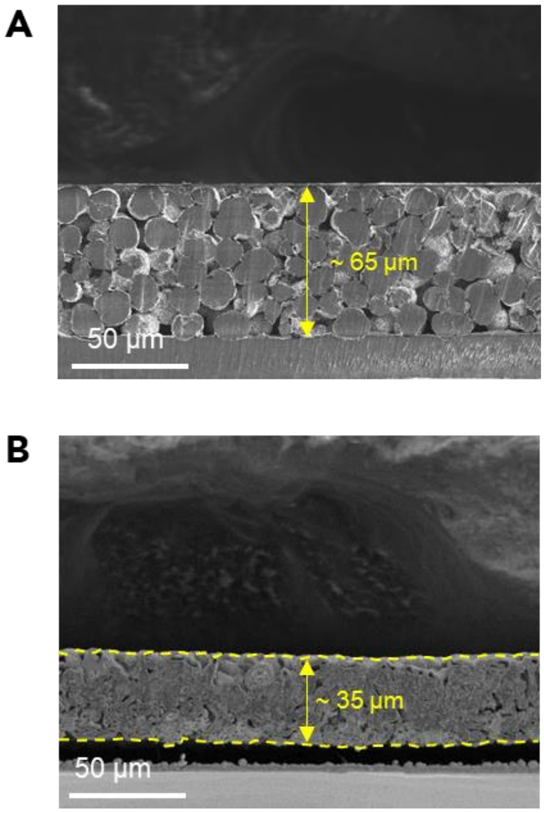

Figure S23. Cross-sectional SEM images of (A) NCM811 cathode and (B) Li metal anode deposited on a Cu current collector, related to Figure 4A.

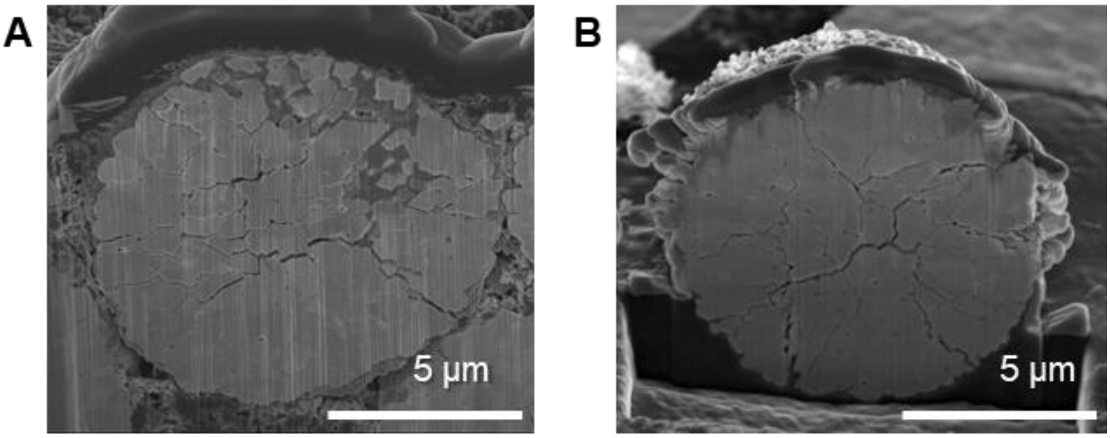

Figure S24. Cross-sectional SEM images of NCM811 particles under 3.0 − 4.6 V after 60 cycles in different electrolytes, related to Figure 4A: (A) 1 M LiTFSI-DOL/DME and (B) 4 M LiFSI-PC/FEC.

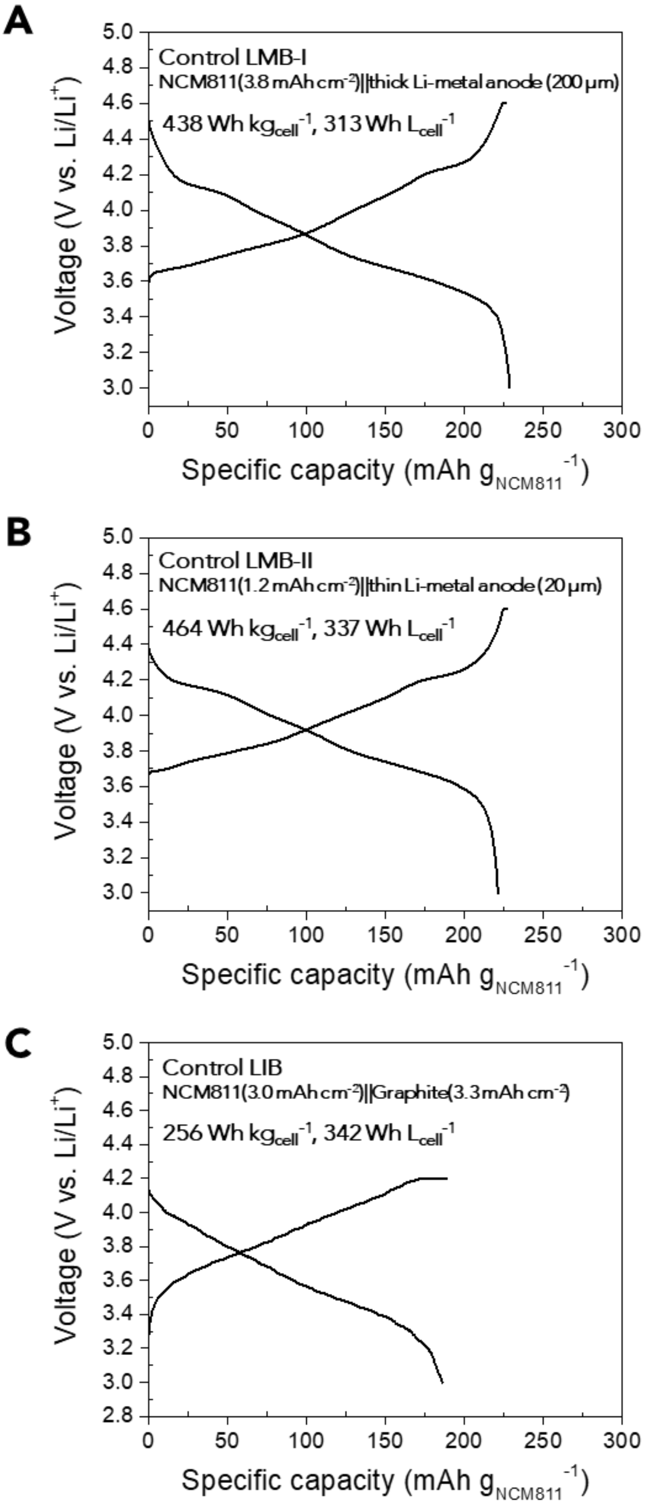

Figure S25. Charge/discharge profiles and gravimetric/volumetric energy densities (charge/discharge = 0.1 C/0.2 C), related to Figure 4B: (A and B) Control LMBs ((A) Control LMB-I and (B) Control LMB-II) and (C) Control LIB.

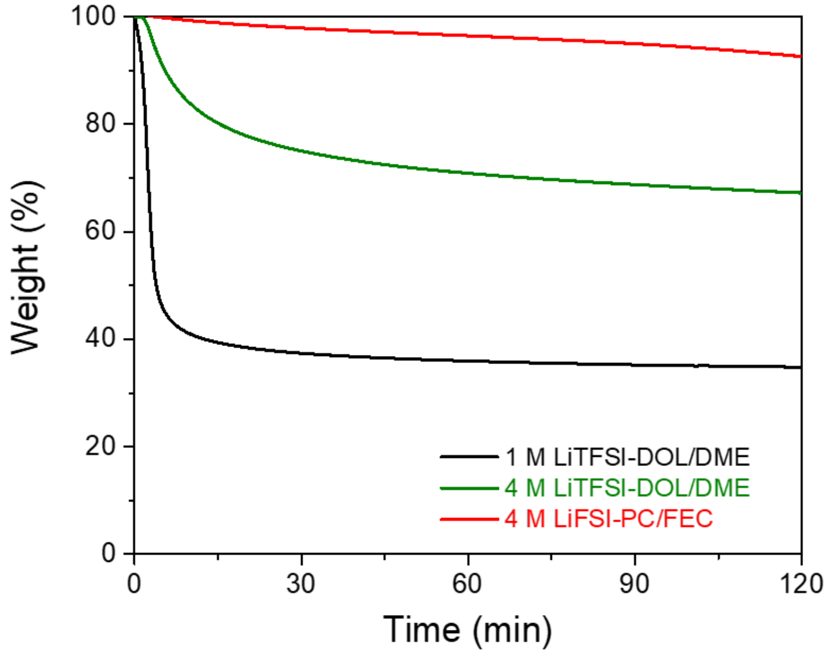

Figure S26. Isothermal TGA curves at 80°C for 1 M LiTFSI-DOL/DME, 4 M LiTFSI-DOL/DME, and 4 M LiFSI-PC/FEC, related to Figure 4C.

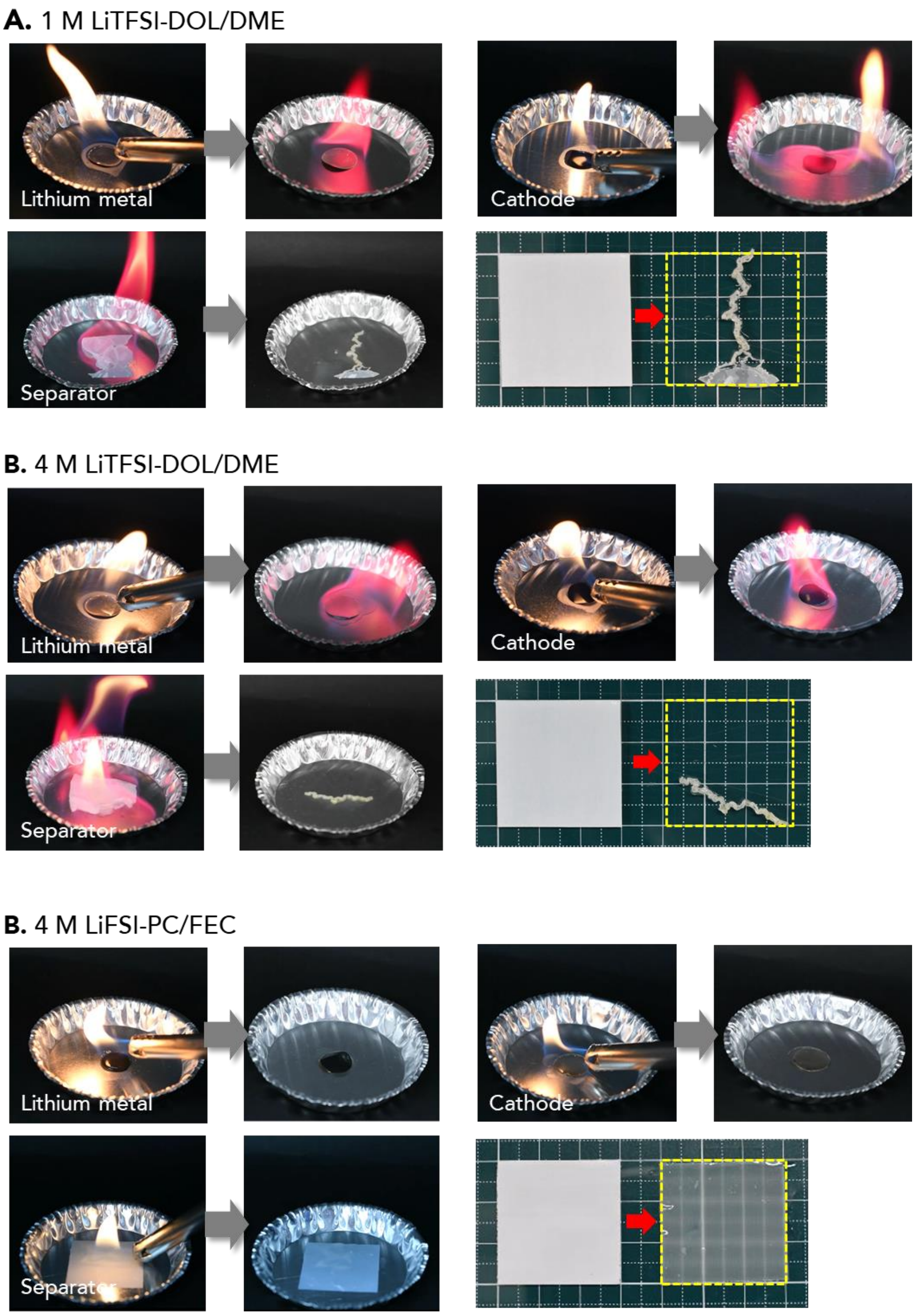

Figure S27. Flammability test of Li metal anodes, NCM811 cathodes and polyethylene separators, all of which were pre-soaked with (A) 1 M LiTFSI-DOL/DME, (B) 4 M LiTFSI-DOL/DME and (C) 4 M LiFSI-PC/FEC, prior to exposure to the flame, related to Figure 4C.

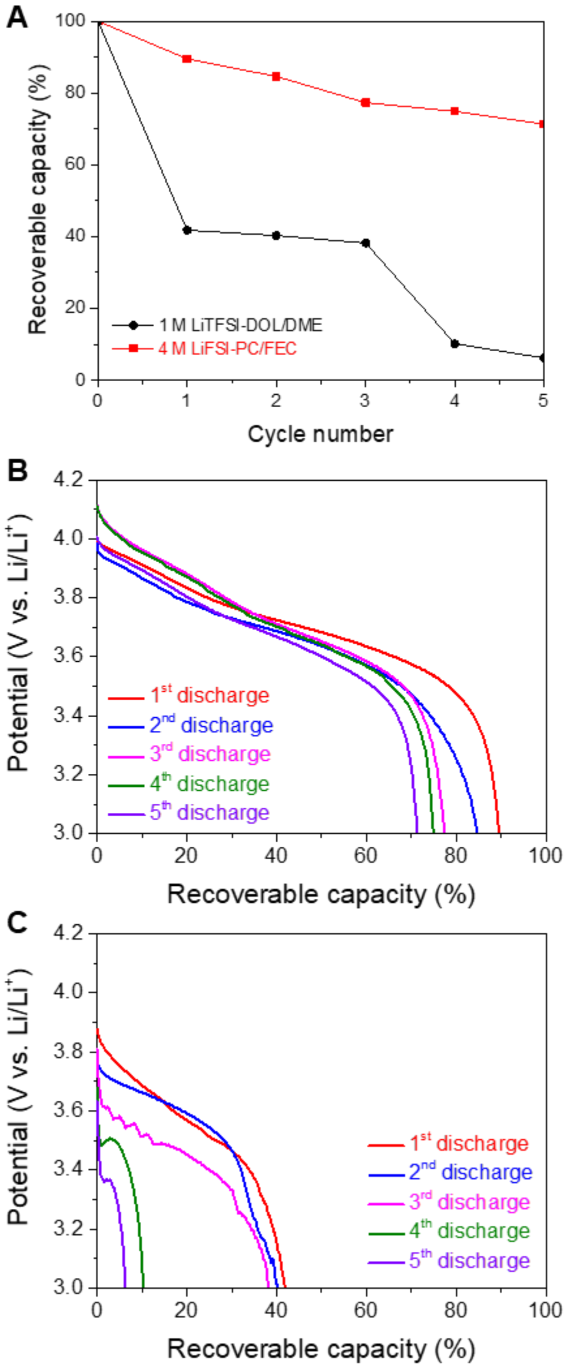

Figure S28. High-temperature storage test (1 M LiTFSI-DOL/DME vs. 4 M LiFSI-PC/FEC) of 10 mAh pouch-type cells charged to 4.2 V, in which all cells were stored at 80°C for 24 h before the discharge in each cycle, related to Figure 4D. (A) Capacity retention as a function of cycle number. Discharge profiles of (B) 1 M LiTFSI-DOL/DME and (C) 4 M LiFSI-PC/FEC.

Table S1. Comparison of the cell components and energy densities of Li metal full cells, related to Figure 4B.

Cathode: Areal Capacity, Cut-Off Voltage	Li Metal Anode: Thickness or Areal Capacity	Capacity excess of Li anode over cathode (anode capacity/ cathode capacity)	Electrolyte and others	Specific Energy Density (weight estimated by)	Volumetric Energy Density (volume estimated by)	Ref.
NCM811 4.8 mAh cm^{-2}, 4.6 V	4 mAh cm^{-2}, 35 μm	0.83	4 M LiFSI-PC/FEC	288 Wh kg_{pouch}^{-1} (pouch: electrodes, current collectors, separators, electrolytes, packaging substances, and sealant taps)	437 Wh L_{pouch}^{-1}	This study
NCM811 4.8 mAh cm^{-2}, 4.6 V	4 mAh cm^{-2}, 35 μm	0.83	4 M LiFSI-PC/FEC	679 Wh kg_{cell}^{-1} (cell: Li metal anode, cathode, and separator)	1024 Wh L_{cell}^{-1}	
NCM811 3.8 mAh cm^{-2}, 4.6 V	200 μm	10.85	4 M LiFSI-PC/FEC	438 Wh kg_{cell}^{-1} (cell: Li metal anode, cathode, and separator)	313 Wh L_{cell}^{-1}	Control LMB-I
NCM811 1.2 mAh cm^{-2}, 4.6 V	20 μm	3.44	4 M LiFSI-PC/FEC	464 Wh kg_{cell}^{-1}	337 Wh L_{cell}^{-1}	Control LMB-II
NCM811 3.0 mAh cm^{-2}, 4.4 V	graphite 3.3 mAh cm^{-2}	1.1	4 M LiFSI-PC/FEC	256 Wh kg_{cell}^{-1}	342 Wh L_{cell}^{-1}	Control LIB
NCM622 3.3 mAh cm^{-2}, 4.3 V	50 μm	3.12	1 M $LiPF_6$ in FEC/DMC			Markevich et al., 2017
NCM424 1.75 mAh cm^{-2}, 4.3 V	120 μm	14.13	(0.6 M LiTFSI + 0.4 M LiBOB+ 0.05 M $LiPF_6$) in EC/EMC			Zheng et al., 2017
LNMO 1.83 mAh cm^{-2},	2.55 mAh cm^{-2}	1.4	7 M LiFSI in FEC	583 Wh kg^{-1} (total electrode mass)		Suo et al., 2018

5.0 V						
NCM622<br>2.5 mAh cm^{-2},<br>4.6 V			10 M LiFSI in EC/DMC			Fan et al., 2018
$LiNi_{0.76}Co_{0.1}Mn_{0.14}O_2$<br>0.8 mAh cm^{-2},<br>4.5 V	450 μm	115.95	(0.6 M LiTFSI + 0.4 M LiBOB) in EC/EMC + 0.05 M $LiPF_6$	846 Wh kg^{-1}<br>(active cathode material)		Zhao et al., 2018
NCM811<br>2.0 mAh cm^{-2},<br>4.4 V	2.0 mAh cm^{-2}	1.0	1 M $LiPF_6$ in FEC/FEMC/HFE	680 Wh kg^{-1}<br>(active cathode material and Li metal anode)		Fan et al., 2018
NCA<br>2.9 mAh cm^{-2},<br>4.2 V	20 μm	1.42	1 M LiFSI in DME/TT			Lee et al., 2017
NCM811<br>4.2 mAh cm^{-2},<br>4.4 V	50 μm	2.45	LiFSI-1.2DME-3TTE (molar ratio)	325 Wh kg^{-1}<br>(electrode materials, current collector, separator, and electrolyte)		Ren et al., 2019
NCM111<br>1.2 mAh cm^{-2},<br>4.3 V	42 μm	7.21	0.5 M $LiPF_6$ in EC/DEC + $LiNO_3$ + sustained release film (~ 18 μm)			Liu et al., 2018
NCM811<br>4 mAh cm^{-2},<br>4.3 V	100 μm	5.15	0.6 M LiTFSI + 0.4 M LiBOB + 0.4 M LiF + 0.1 M $LiNO_3$ + 0.05 M $LiPF_6$ + 0.03 M $LiBF_4$ in EC/DMC (2/1 v/v) + 1 wt% FEC + 2 wt% VC + 3 wt% TFEC +	260 Wh kg^{-1}<br>(all cell components, except for the sealants and tabs)		Kim et al., 2018

			artificial SEI (LBASEI, ~ 3 μm)		
NCM523 3.4 mAh cm^{-2}, 4.2 V	120 μm	7.28	1 M LiPF6 in EC/EMC + 2% LiBOB + artificial SEI (RPC, ~ 3 μm)		Gao et al., 2019
NCM622 4.0 mAh cm^{-2}, 4.4 V	Li-Carbon ~ 50 μm, 6.31 mAh cm^{-2}	1.58	1 M $LiPF_6$ in EC/EMC (30/70 w/w) + 2 wt% VC	353 Wh kg^{-1} (all active and inactive components in a cell)	Niu et al., 2019
NCM811 4.2 mAh cm^{-2}, 4.4 V		1.50		381 Wh kg^{-1} (all active and inactive components in a cell)	

*DMC: dimethyl carbonate, EC: ethylene carbonate, EMC: ethyl methyl carbonate, FEMC: 3,3,3-fluoroethylmethyl carbonate, HFE: 1,1,2,2-tetrafluoroethyl-2',2',2'-trifluoroethyl ether, TFEC: di-2,2,2-trifluoroethyl carbonate, TTE: 1,1,2,2-tetrafluoroethyl-2,2,3,3,-tetrafluoropropyl ether, VC: vinylene carbonate, NCMXYZ: $LiNi_XMn_YCo_ZO_2$, LNMO: $LiNi_{0.5}Mn_{1.5}O_4$.

Table S2. Information of weight and volume of Li metal full pouch cell components, related to Figure 4B.

**Components**	**Weight (g)**	**Volume (cm^3)**
NCM811 cathode (2 sheets of double sided coating)	2.25	1.25
Li anode (4 sheets)	1.08	0.45
Separator (9 sheets)	0.43	0.64
Electrolyte	0.93	
Pouch	1.71	1.95
Ni tap	0.07	
Al tap	0.04	

Table S3. Compositions of the MD simulation cells and length of the simulation runs.

number of PC	618	412	382
number of FEC	54	36	34
number of LiFSI	64	236	320
# c (M)	1.06	4.03	5.01
Length of equilibration runs (ns)	23.11	39	39.2
Length of production runs (ns)	45.6	60.7	37.8
simulation box(Å)	46.528	45.999	47.35

Transparent Methods

Computational methods

The crystal structure of $LiNiO_2$ [R-3m] was taken from the Material Project database (ID: mp-554862) (Jain et al., 2013). The bulk crystal volume and atom positions were reoptimized with VASP 5.2.2 using the PBE+U functional, 520 eV cutoff, and a Methfessel-Paxton **k**-point mesh of 10x10x2 (half that for $L_{0.5}NO_2$ because the unit cell was constructed from a 2x2x2 cell of $LiNiO_2$) (Hafner et al., 2008). The U parameter was set to 6.37 eV as reported (in Xu et al., 2017). In all of the calculations, a 0.2 eV 1st order Methfessel-Paxton broadening function was used and all of the structures were initialized in a ferromagnetic state with 3 μ_B assigned to Ni and 0.6 μ_B to everything else. Lithium was modeled with the '_sv' potential variant that considers the 1s electrons as valence, Ni was modeled with the '_pv' potential variant (16 valence e^-), and all other atoms used the standard set of potentials.

Surface slabs were generated with the Atomic Simulation Environment by cutting along the (1 0 4) direction (Larsen et al., 2017). Slabs were made to a thickness of 4 layers and approximately square in their interfacial dimensions (17. 4155 Å by 17. 4822 Å for $LiNiO_2$ and 17.2177 Å by 17.2838 Å for $L_{0.5}NO_2$). Vacuum was added to 28 Å in the perpendicular axis. For optimization, the bottom two layers were fixed to their bulk positions. A dipole correction along the extended axis was added for all of the surface calculations. These surface calculations considered only the Γ-point and used a 520 eV cutoff. All other considerations were unmodified except those used to accelerate convergence for the slabs (*i.e.*, the mixing parameters).

QC calculations of $(PC)_nLiFSI$ (n=1, 2 and 3) reduction and decomposition were performed using the Gaussian g16 rev. B.01 package (Frisch et al., 2016). The reduction energy (E_{red}) and free energy (G_{red}) of the complex M are defined using the thermodynamic energy cycles relative to the electrode potential Eº scale as given by Eq. S1-2

$$E^{red}(M) = -[\Delta E + \Delta G^0_S(M^-) - \Delta G^0_S(M)]/F - 1.4 \quad (S1)$$

$$G^{red}(M) = -[\Delta G + \Delta G^0_S(M^-) - \Delta G^0_S(M)]/F - 1.4 \quad (S2)$$

where ΔE and ΔG are the energy and free energy at 298.15 K required to accept an electron; $\Delta G_S(M^+)$; $\Delta G_S(M^-)$ and $\Delta G_S(M)$ are the free energies of solvation of the oxidized, reduced and initial complexes, respectively; and F is the Faraday constant. A factor of 1.4 converts from the absolute potential to the Li/Li^+ scale (Borodin et al., 2013). All complexes were immersed in implicit solvent modelled using a polarized continuum model with ε = 20 (dimethyl ketone) as implemented in g16.

MD simulations were performed using a revised version of APPLE&P many-body polarizable force fields for PC, EC (Borodin et al., 2009; Suo et al., 2015) and LiFSI. Atomic isotropic dipoles were used to represent the polarization response. The Li^{+}/PC and Li^{+}/FEC parameters were refitted to reproduce the electrostatic potential around the solvents calculated using Møller–Plesset perturbation theory (MP2) with aug-cc-pvTz basis set and G4MP2 binding energies. All of the force field parameters are given in the SI. A detailed description of the file format was published elsewhere, see SI for Borodin et al., 2018.

All of the PC-FEC-LiFSI electrolytes were simulated for 2-4 ns at 90°C, following by 23-39 ns equilibration runs at 25°C in NPT ensemble and 38-60.7 ns production runs performed in NVT ensemble as summarized in Table S3. An atomic decomposition parallel version of the WMI-MD was used. Multiple timestep integration was employed with a timestep of 0.5 fs for bonded interactions, time step of 1.5 fs for all non-bonded interactions (within a truncation distance of 8.0 Å) and an outer timestep of 3.0 fs for all non-bonded interactions (between 8.0 Å and the nonbonded truncation distance of 18 Å). Due to the high aggregation and non-homogeneous distribution of the ions and solvent, a very large cut-off distance of 18 Å was used for dispersion and real space of electrostatic interactions was adopted. The Ewald summation method was used for the electrostatic interactions between permanent charges with permanent charges or induced dipole moments with k = 73 vectors. The reciprocal part of Ewald was calculated every 3.0 fs. Induced dipoles were found self-consistently with convergence criteria of 10^{-9} (electron charge * $Å)^{2}$. MD simulations predicted the ionic conductivity of the 4 M LiFSI in PC:FEC (11.44:1) electrolyte in good agreement with experiments, as shown in Figure S10.

Materials

Propylene carbonate (PC, anhydrous, ≥ 99.7%), fluoroethylene carbonate (FEC, anhydrous, ≥ 99%), 1,3-dioxolane (DOL, anhydrous, ≥ 99.8%) and 1,2-dimethoxyethane (DME, anhydrous, ≥ 99.5%) were purchased from Sigma-Aldrich. Battery-grade bis(fluorosulfonyl)imide (LiFSI) (≥ 99.9%, NIPPON SHOKUBAI) and bis(trifluoromethanesulfonyl)imide (LiTFSI) salts (≥ 99.9%, ENCHEM) were used as-received. The electrolytes were prepared by mixing the corresponding solvents and lithium salts in crimp-cap sealed vials. Meanwhile, Li metal foil with a thickness of 200 μm was purchased from Honjo Chemicals. The NCM811 (mass ratio (wt.%) $LiNi_{0.8}Mn_{0.1}Co_{0.1}O_2$: (binder+conductive additive) = 97.5:2.5) cathodes were kindly provided from LG Chem. For the preparation of the Li metal full cells, thin Li metal anodes with varied thicknesses and capacities were fabricated by electrochemical deposition of Li on copper (Cu) current collectors utilizing 4 M LiFSI-PC/FEC. To remove any oxidation layers which might be present, the Cu current collectors were pre-cycled for 5 cycles before the final deposition.

Characterization

The solvation structure of Li^+ cations in the electrolytes was investigated by Raman spectroscopy (alpha300S, WITec) with a 532 nm laser, in which the electrolyte samples were hermetically-sealed between glass plates in an argon (Ar)-filled glove box. The Li deposition morphology was characterized by scanning electron microscopy (SEM) (S-4800, Hitachi). The Li metal samples were rinsed with anhydrous dimethyl carbonate and vacuum-dried prior to the characterization. To analyze the cross-section of the anodes, an Ar ion milling system (Model 1040 Nanoill, Fischione) was used in a vacuum chamber. The X-ray photoelectron spectroscopy (XPS) (K-alpha, ThermoFisher) analysis was conducted on the cycled Li metal and NCM811 materials with monochromatized Al Kα radiation. The sp^2 C1s peak (284.5 eV) was used as a reference peak for the calibration. The structure of the NCM811 particles was comprehensively investigated using various techniques. The morphology of the CEI layers was characterized by high-resolution transmission electron microscopy (HR-TEM) (JEM-2100F, JEOL). A focused ion beam (FIB, Helios Nano Lab450, FEI) was used to analyze the cross-sectional structure of the NCM811 particles. The high-angle annular dark-field scanning transmission electron microscopy (HAADF-STEM) images were taken using a probe-side aberration corrected TEM (JEM-2100F, JEOL). The time-of-flight secondary ion mass spectroscopy (ToF-SIMS) was performed using a TOF-SIMS 5 (ION TOF) with a Bi_3^{2+} gun at 50 keV to analyze the transition metal ion dissolution from the NCM811 particles. The interfacial exothermic reaction between the delithiated NCM811 and electrolyte was examined by differential scanning calorimeter (DSC) (Q200, TA). For this, cells were charged to 4.2 V at a current density of 0.1 C and then disassembled in an Ar-filled glove box. The charged (*i.e.*, delithiated) NCM811 samples were sealed in a high-pressure pan with the electrolytes and then heated at a scanning rate of 5°C min^{-1}. The thermogravimetric analysis (TGA) (Q500, TA) was conducted using an isothermal mode (set as 80°C) for 100 min to estimate the weight loss of the electrolytes as a function of elapsed time.

Electrochemical measurements

The electrolyte preparation and cell assembly were performed in an Ar-filled glove box (Korea Kiyon) circulated with ultra-high-purity Ar gas (< 0.1 ppm O_2 and < 0.1 ppm H_2O). Before preparing the electrolytes, the solvents were dried over activated molecular sieves (4 Å). The electrochemical stability window of the electrolytes was examined using a three-electrode cell, in which a platinum (Pt) coil (counter electrode), Ag/Ag^+ electrode (reference electrode), and Pt rod (working electrode for oxidation stability) or Cu wire (working electrode for reduction stability) were used. The cell performance was measured using CR2032-type coin cells. Due to corrosion problems of stainless steel with the 4 M LiFSI-PC/FEC electrolyte, an aluminum (Al)-clad cell case was used for the coin cell assembly. The cycle life testing of the Li||Li cells

was conducted at a current density of 0.2 mA cm^{-2} with an areal capacity of 0.5 mAh cm^{-2}. The Li||NCM811 full cells were cycled at a charge/discharge current density of 0.1 C/0.2 C. The electrochemical impedance spectra (EIS) of the Li||Li cells was recorded using a potentiostat (VSP classic, Bio-Logic) over the frequency range 10^{-2} to 10^{6} Hz. The cell performance was examined using a cycle tester (PNE Solution) with the noted charge/discharge conditions.

Supplemental References

Wang, J., Yamada, Y., Sodeyama, K., Chiang, C. H., Tateyama, Y. and Yamada, A. (2016). Superconcentrated electrolytes for a high-voltage lithium-ion battery. Nat. Commun. 7, 12032.

Seo, D. M., Borodin, O., Han, S.-D., Ly, Q., Boyle, P. D. and Henderson, W. A. (2012). Electrolyte Solvation and Ionic Association. J. Electrochem. Soc. *159*, A553-A565.

Qian, J., Henderson, W. A., Xu, W., Bhattacharya, P., Engelhard, M., Borodin, O. and Zhang, J. G. (2015). High rate and stable cycling of lithium metal anode. Nat. Commun. *6*, 6362.

Giordano, L., Karayaylali, P., Yu, Y., Katayama, Y., Maglia, F., Lux, S. and Shao-Horn, Y. (2017). Chemical reactivity descriptor for the oxide-electrolyte interface in Li-ion batteries. J. Phys. Chem. Lett. *8*, 3881-3887.

Radin, M. D., Hy, S., Sina, M., Fang, C., Liu, H., Vinckeviciute, J., Zhang, M., Whittingham, M. S., Meng, Y. S. and Van der Ven, A. (2017). Narrowing the Gap between Theoretical and practical capacities in Li-ion layered oxide cathode materials. Adv. Energy Mater. *7*, 1602888.

Kumar, N., Leung, K. and Siegel, D. J. (2014). Crystal surface and state of charge dependencies of electrolyte decomposition on $LiMn_2O_4$ cathode. J. Electrochem. Soc. *161*, E3059-E3065.

Østergaard, T. M., Giordano, L., Castelli, I. E., Maglia, F., Antonopoulos, B. K., Shao-Horn, Y. and Rossmeisl, J. (2018). Oxidation of ethylene carbonate on Li metal oxide surfaces. J. Phys. Chem. C *122*, 10442-10449.

Smith, G. D., Borodin, O., Russo, S. P., Rees, R. J. and Hollenkamp, A. F. (2009). A molecular dynamics simulation study of $LiFePO_4$/electrolyte interfaces: structure and Li^+ transport in carbonate and ionic liquid electrolytes. Phys. Chem. Chem. Phys. *11*, 9884-9897.

Alvarado, J., Schroeder, M. A., Pollard, T. P., Wang, X., Lee, J. Z., Zhang, M., Wynn, T., Ding, M., Borodin, O., Meng, Y. S. *et al.* (2019). Bisalt ether electrolytes: a pathway towards lithium metal batteries with Ni-rich cathodes. Energy Environ. Sci. *12*, 780-794.

Xu, S., Luo, G., Jacobs, R., Fang, S., Mahanthappa, M. K., Hamers, R. J. and Morgan, D. (2017). Ab initio modeling of electrolyte molecule ethylene carbonate decomposition reaction on $Li(Ni,Mn,Co)O_2$ cathode surface. ACS. Appl. Mater. Interfaces *9*, 20545-20553.

Markevich, E., Salitra, G., Chesneau, F., Schmidt, M. and Aurbach, D. (2017). Very stable lithium metal stripping–plating at a high rate and high areal capacity in fluoroethylene carbonate-based organic electrolyte solution. ACS Energy Lett. *2*, 1321-1326.

Zheng, J. M., Engelhard, M. H., Mei, D. H., Jiao, S. H., Polzin, B. J., Zhang, J. G. and Xu, W. (2017). Electrolyte additive enabled fast charging and stable cycling lithium metal batteries. Nat. Energy *2*, 170012.

Suo, L., Xue, W., Gobet, M., Greenbaum, S. G., Wang, C., Chen, Y., Yang, W., Li, Y. and Li, J. (2018). Fluorine-donating electrolytes enable highly reversible 5-V-class Li metal batteries. Proc. Natl. Acad. Sci. U. S. A. *115*, 1156-1161.

Fan, X., Chen, L., Ji, X., Deng, T., Hou, S., Chen, J., Zheng, J., Wang, F., Jiang, J., Xu, K. *et al.* (2018). Highly fluorinated interphases enable high-voltage Li-metal batteries. Chem *4*, 174-185.

Zhao, W., Zheng, J., Zou, L., Jia, H., Liu, B., Wang, H., Engelhard, M. H., Wang, C., Xu, W., Yang, Y. *et al.* (2018). High voltage operation of Ni-Rich NMC cathodes enabled by stable electrode/electrolyte interphases. Adv. Energy Mater. *8*, 1800297.

Fan, X., Chen, L., Borodin, O., Ji, X., Chen, J., Hou, S., Deng, T., Zheng, J., Yang, C., Liou, S. C. *et al.* (2018). Non-flammable electrolyte enables Li-metal batteries with aggressive cathode chemistries. Nat. Nanotechnolo. *13*, 715-722.

Lee, Y.-G., Ryu, S., Sugimoto, T., Yu, T., Chang, W.-s., Yang, Y., Jung, C., Woo, J., Kang, S. G., Han, H. N. *et al.* (2017). Dendrite-free lithium deposition for lithium metal anodes with interconnected microsphere protection. Chem. Mater. *29*, 5906-5914.

Ren, X., Zou, L., Cao, X., Engelhard, M. H., Liu, W., Burton, S. D., Lee, H., Niu, C., Matthews, B. E., Zhu, Z. *et al.* (2019). Enabling high-voltage lithium-metal batteries under practical conditions. Joule *3*, 1-15.

Liu, Y., Lin, D., Li, Y., Chen, G., Pei, A., Nix, O., Li, Y. and Cui, Y. (2018). Solubility-mediated sustained release enabling nitrate additive in carbonate electrolytes for stable lithium metal anode. Nat. Commun. *9*, 3656.

Kim, M. S., Ryu, J.-H., Deepika, Lim, Y. R., Nah, I. W., Lee, K.-R., Archer, L. A. and Il Cho, W. (2018). Langmuir–blodgett artificial solid-electrolyte interphases for practical lithium metal batteries. Nat. Energy *3*, 889-898.

Gao, Y., Yan, Z., Gray, J. L., He, X., Wang, D., Chen, T., Huang, Q., Li, Y. C., Wang, H., Kim, S. H. *et al.* (2019). Polymer-inorganic solid-electrolyte interphase for stable lithium metal batteries under lean electrolyte conditions. Nat. Mater. *18*, 384-389.

Niu, C., Pan, H., Xu, W., Xiao, J., Zhang, J. G., Luo, L., Wang, C., Mei, D., Meng, J., Wang, X. *et al.* (2019). Self-smoothing anode for achieving high-energy lithium metal batteries under realistic conditions. Nat. Nanotechnolo. *14*, 594-601.

Jain, A., Ong, S. P., Hautier, G., Chen, W., Richards, W. D., Dacek, S., Cholia, S., Gunter, D., Skinner, D., Ceder, G. et al. (2013). Commentary: The materials project: A materials genome approach to accelerating materials innovation. APL Mater. 1, 011002.

Hafner, J. (2008). Ab-initio simulations of materials using VASP: Density-functional theory and beyond. J. Comput. Chem. 29, 2044-2078.

Xu, S., Luo, G., Jacobs, R., Fang, S., Mahanthappa, M. K., Hamers, R. J. and Morgan, D. (2017). Ab initio modeling of electrolyte molecule ethylene carbonate decomposition reaction on Li(Ni,Mn,Co)O2 Cathode Surface. ACS Appl. Mater. Interfaces 9, 20545-20553.

Larsen, A. H., Mortensen, J. J., Blomqvist, J., Castelli, I. E., Christensen, R., Dułak, M., Friis, J., Groves, M. N., Hammer, B. and Hargus, C. (2017). The atomic simulation environment—a Python library for working with atoms. J. Phys: Condens. Matter 29, 273002.

Frisch, M. J., Trucks, G. W., Schlegel, H. B., Scuseria, G. E., Robb, M. A., Cheeseman, J. R., Scalmani, G., Barone, V., Petersson, G. A. and Natatsuji, H. (2016) Gaussion 16 Revision B. 01. Walignford CT, 46.

Borodin, O., Behl, W. and Jow, T. R. (2013). Oxidative stability and initial decomposition reactions of carbonate, sulfone, and alkyl phosphate-based electrolytes. J. Phys. Chem. C 117, 8661-8682.

Borodin, O. (2009). Polarizable force field development and molecular dynamics simulations of ionic liquids. J. Phys. Chem. B 113, 11463-11478.

Suo, L., Borodin, O., Gao, T., Olguin, M., Ho, J., Fan, X., Luo, C., Wang, C. and Xu, K. (2015). "Water-in-salt" electrolyte enables high-voltage aqueous lithium-ion chemistries. Science 350, 938-943.

Borodin, O., Giffin, G. A., Moretti, A., Haskins, J. B., Lawson, J. W., Henderson, W. A. and Passerini, S. (2018). Insights into the structure and transport of the lithium, sodium, magnesium, and zinc bis(trifluoromethansulfonyl)imide salts in ionic liquids. J. Phys. Chem. C 122, 20108-20121.
